# Principles of amyloplast replication in the ovule integuments of *Arabidopsis thaliana*

**DOI:** 10.1093/plphys/kiae314

**Published:** 2024-06-03

**Authors:** Makoto T Fujiwara, Yasushi Yoshioka, Yusuke Kazama, Tomonari Hirano, Yasuo Niwa, Takashi Moriyama, Naoki Sato, Tomoko Abe, Shigeo Yoshida, Ryuuichi D Itoh

**Affiliations:** Nishina Center and Plant Functions Laboratory (Disbanded in March 2004), RIKEN, Wako, Saitama 351-0198, Japan; Department of Biology, Graduate School of Science and Technology, Sophia University, Kioicho, Chiyoda 102-8554, Japan; College of Arts and Sciences, University of Tokyo, Komaba, Tokyo 153-8902, Japan; Graduate School of Science, Nagoya University, Furo-cho, Nagoya 464-8602, Japan; Nishina Center and Plant Functions Laboratory (Disbanded in March 2004), RIKEN, Wako, Saitama 351-0198, Japan; Nishina Center and Plant Functions Laboratory (Disbanded in March 2004), RIKEN, Wako, Saitama 351-0198, Japan; Laboratory of Plant Cell Technology, University of Shizuoka, Yada, Shizuoka 422-8526, Japan; College of Arts and Sciences, University of Tokyo, Komaba, Tokyo 153-8902, Japan; College of Arts and Sciences, University of Tokyo, Komaba, Tokyo 153-8902, Japan; Nishina Center and Plant Functions Laboratory (Disbanded in March 2004), RIKEN, Wako, Saitama 351-0198, Japan; Nishina Center and Plant Functions Laboratory (Disbanded in March 2004), RIKEN, Wako, Saitama 351-0198, Japan; Department of Chemistry, Biology and Marine Science, Faculty of Science, University of the Ryukyus, Senbaru 1, Nishihara, Okinawa 903-0213, Japan

## Abstract

Plastids in vascular plants have various differentiated forms, among which amyloplasts are crucial for starch storage and plant productivity. Despite the vast knowledge of the binary-fission mode of chloroplast division, our understanding of the replication of non-photosynthetic plastids, including amyloplasts, remains limited. Recent studies have suggested the involvement of stromules (stroma-filled tubules) in plastid replication when the division apparatus is faulty. However, details of the underlying mechanism(s) and their relevance to normal processes have yet to be elucidated. Here, we developed a live analysis system for studying amyloplast replication using Arabidopsis (*Arabidopsis thaliana*) ovule integuments. We showed the full sequence of amyloplast development and demonstrated that wild-type amyloplasts adopt three modes of replication, binary fission, multiple fission, and stromule-mediated fission, via multi-way placement of the FtsZ ring. The *minE* mutant, with severely inhibited chloroplast division, showed marked heterogeneity in amyloplast size, caused by size-dependent but wild-type modes of plastid fission. The dynamic properties of stromules distinguish the wild-type and *minE* phenotypes. In *minE* cells, extended stromules from giant amyloplasts acquired stability, allowing FtsZ ring assembly and constriction, as well as the growth of starch grains therein. Despite hyper-stromule formation, amyloplasts did not proliferate in the *ftsZ* null mutant. These data clarify the differences between amyloplast and chloroplast replication and demonstrate that the structural plasticity of amyloplasts underlies the multiplicity of their replication processes. Furthermore, this study shows that stromules can generate daughter plastids via the assembly of the FtsZ ring.

## Teaser

Live analysis of the Arabidopsis outer ovule integument reveals the regulatory basis of amyloplast replication is complex and differs from that of chloroplast replication.

## Introduction

Understanding the mechanisms of plastid replication is crucial to address how the identity and the activity of plant cells are maintained. Plastids are double-membrane-bound organelles, and the primary plastid type is the chloroplast, which is replicated by symmetric binary fission (Possingham and Lawrence 1983; Leech and Pyke 1988). Intensive studies over the last quarter century have unraveled key molecular players for chloroplast division (Osteryoung and Pyke 2014). In the model plant Arabidopsis (*Arabidopsis thaliana*), the initiation of chloroplast division involves stromal tubulin-like proteins FtsZ1/2 (Osteryoung and Vierling 1995; Osteryoung et al. 1998). FtsZ1/2 assemble into a ring structure on the stromal surface of the inner-envelope membrane at mid-chloroplast before and during chloroplast constriction (Vitha et al. 2001; McAndrew et al. 2001). ARC6, an integral inner-envelope protein (Vitha et al. 2003), facilitates the FtsZ ring assembly and permits further assembly of a cytoplasmic dynamin-related protein ARC5 (Gao et al. 2003) at the division site, via the activities of the outer-envelope proteins PDV1/2 (Miyagishima et al. 2006; Glynn et al. 2008). It is also known that MinE is an indispensable factor for chloroplast division (Itoh et al. 2001; Fujiwara et al. 2008). MinE is targeted to stroma and mediates mid-chloroplast placement of the FtsZ ring in cooperation with MinD, MCD1, and ARC3 (summarized by Miyagishima et al. 2011; Chen et al. 2018). It has been shown that these proteins are derived from mixed evolutionary origins, but act under strict interrelationships to achieve well-ordered chloroplast fission processes. Studies of plastid division coordinations in plants, which are now being undertaken in several model systems, may provide profound insights into the development, inheritance, and evolution of plastids.

In vascular plants, plastids comprise a group of morphologically diverse organelles. In addition to chloroplasts, non-photosynthetic (non-green) plastids, such as chromoplasts, leucoplasts, amyloplasts, etioplasts, and elaioplasts, have been identified in a broad array of tissues by electron microscopy (Kirk and Tilney-Bassett 1978; Thomson and Whatley 1980; Pyke 1999; Wise 2007). Many of these plastids store metabolites or nutrients and serve essential roles in plant productivity. As a typical example, amyloplasts are specified organelles in accumulation of starch grains. Amyloplasts arise by differentiation and proliferate in tubers, endosperms, stem endodermis, root tip columella, and so on (Thomson and Whatley 1980; Possingham and Lawrence 1983; Kuroiwa et al. 1998). Non-photosynthetic plastids also exhibit unique morphological traits. A prominent of them includes the development of thin envelope membrane-derived protrusions, termed stromules (stroma-filled tubules) (Köhler and Hanson 2000). Stromules have highly mobile activities, showing extension, retraction, and branching, and are preferentially activated in non-photosynthetic tissues and/or in response to various stresses (Gray et al. 2001; Kwok and Hanson 2004; Natesan et al. 2005; Pyke 2009; Hanson and Hines 2018; Erickson and Schattat 2018; Hanson and Conklin 2020; Mathur 2012; Mathur 2021). Nevertheless, the physiological importance of stromules remains elusive. Since the early 2000s, non-photosynthetic plastid and stromule morphologies have been analyzed using chloroplast division mutants of tomato *suffulta* (Forth and Pyke 2006), and Arabidopsis *arc3*, *arc5*, *arc6* (Holzinger et al. 2008; Schattat et al. 2012; Fujiwara et al. 2018), *crumpled leaf* (Chen et al. 2009), *minE* (Kojo et al. 2009; Fujiwara et al. 2015), and *parc6* (Itoh et al. 2018; Ishikawa et al. 2020). These studies generally detected the activation of stromules in selected non-photosynthetic tissues of the mutants. It was proposed (Forth and Pyke 2006; Holzinger et al. 2008; Chen et al. 2009; Fujiwara et al. 2015) that stromule formation contributes to the proliferation of chromoplasts, leucoplasts, or other aberrant plastids in these mutants by as yet undefined mechanisms.

Substantial advances have been made in understanding the molecular mechanisms of chloroplast division. However, little is known about those non-photosynthetic plastids. One reason for this might be the scarcity of live model systems that facilitate the characterization of non-photosynthetic plastid division. While the leaf mesophyll chloroplasts have been a primary model for studying the control of plastid division, it is important to analyze how the replication of non-photosythetic plastids is controlled. Arabidopsis is an excellent model plant for gaining insights into the genetic and molecular control of plastid replication. Various kinds of plastid types can be studied by introducing stroma-targeted fluorescent proteins and performing live microscopy (Tirlapur et al. 1999; Hanson and Köhler 2001; Suzuki et al. 2013). Numerous plastid morphogenesis mutants of Arabidopsis, such as *accumulation and replication of chloroplasts* (*arc*) (Pyke 1997; Marrison et al. 1999), have been exploited to study plastid division. Among the non-photosynthetic plastids, amyloplasts have received considerable attention in crop biology (Kirk and Tilney-Bassett 1978; Thomson and Whatley 1980), and their proliferation processes have been well examined at the ultrastructural level (Buttrose 1960; Hoshikawa 1968; Kuroiwa et al. 1998; Sagisaka 2008). Although FtsZ1/2, ARC5, MinD/E, PDV1/2, and ARC6/PARC6 orthologs have been shown to play a role in either amyloplast proliferation or starch grain morphology in potato tuber, rice endosperm, or wheat endosperm (de Pater et al. 2006; Yun and Kawagoe 2009; Yun and Kawagoe 2010; Kamau et al. 2015; Esch et al. 2023; Pfotenhauer et al. 2023), the molecular mechanism of amyloplast division remains unclear. We recently noticed that Arabidopsis outer ovule integuments accumulate amyloplasts during seed development (Western et al. 2000; Windsor et al. 2000) and are useful for studying amyloplast replication, taking advantage of the available transgenic and mutant resources. In this study, using stromule development and FtsZ ring formation as two key events, we found that partly common, but critically distinct, regulatory mechanisms operate between amyloplast and chloroplast replication. Additionally, live imaging analysis uncovered a link between amyloplast replication and stromule formation, which indicates that stromules serve as a platform for FtsZ ring assembly, depending on their stability.

## Results

### Outer ovule integument as a live model system for the analysis of amyloplast replication and development

**Figure 1.**
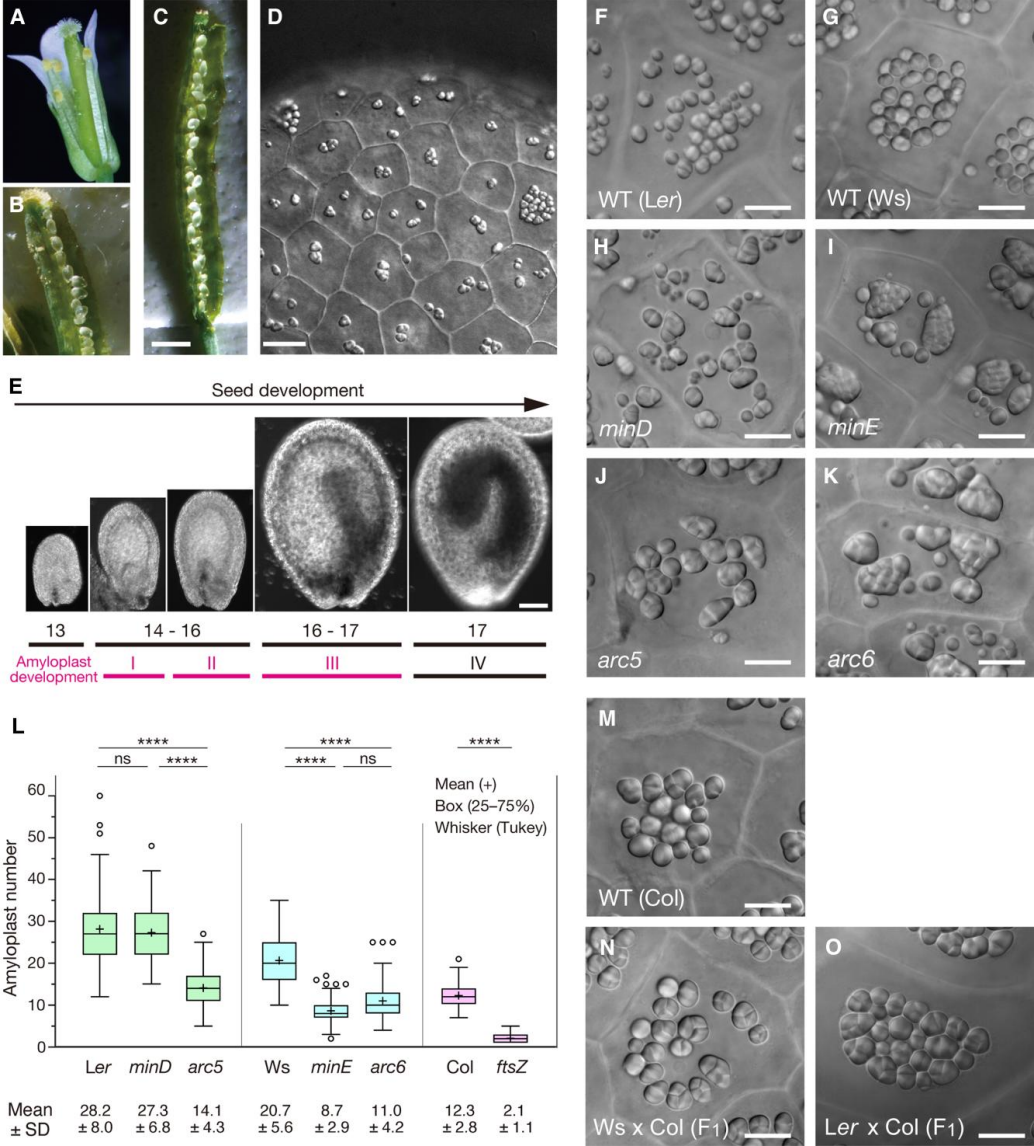
Amyloplast development in the outer ovule integument of wild-type plants and four plastid division mutants of Arabidopsis. **A, B)** Dissection of an open flower of Arabidopsis (**A**) for the extraction of ovules from a pistil **(B)**. **C)** An extended silique with ovules or developing seeds. **D)** Starch grains in the epidermis of an ovule integument. **E)** Comparison of the developmental stages of amyloplast (Phases I–IV; see Fig. 2A) and flower (stages 13–17; Smyth et al. 1990; Schneitz et al. 1995). **F–K)** Terminal phenotypes of amyloplast replication in integument cells of wild-type (WT) **(F, G)**, *minD***(H)**, *minE***(I)**, *arc5***(J)**, and *arc6***(K)** plants. **L)** Boxplots of amyloplast number in mature integument cells. For each sample, 100 cells from three independent plants were observed, and values of mean ± SD are shown. Elements of the boxplot are described in MATERIALS AND METHODS. The differences between plants were analyzed by a nested one-way ANOVA with Tukey's multiple comparison tests (**** *P* < 0.0001). See also Supplementary Fig. S1. **M–O)** Amyloplast phenotypes in integument cells of WT (Col) and its crossed F_1_ plants (the pollen donor: Col) **(N, O)**. Scale bars: 1 mm (**C**), 10 *µ*m **(D, F–K, M–O)**, and 100 *µ*m **(E)**.

Ovule integuments are the precursors of the seed coat. In Arabidopsis, they consist of a double layer of tissues, the outer and the inner integuments, the former of which are two-cell layered and predominantly accumulate starch grains after fertilization (Western et al. 2000; Windsor et al. 2000). We found that the outermost tissue of the outer ovule integuments (alternatively termed as oi2, or epidermis) had potentially advantageous properties for dissecting amyloplast division *in planta* (Fig. 1, A to D): (1) the outer integument cells take a tile form with a relatively constant depth throughout the organ; (2) amyloplasts containing single or compound starch granules (Western et al. 2000; Windsor et al. 2000; Young et al. 2008) can be easily detected in whole-mount live ovules, without tissue destruction, under light microscopy; (3) the development of ovules within pistils and the transdifferentiation of amyloplasts from proplastids in individual ovules proceed in a relatively synchronous manner; and (4) amyloplast proliferation is really active at the early stages of seed development and does not involve cell division (Phases I and II in Fig. 1E; see below). It was speculated that these characteristics would allow for a faithful characterization of amyloplast replication in relation to tissue and organelle development.

### Non-conservative phenotypes of amyloplast replication in Arabidopsis plastid division mutants

**Figure 2.**
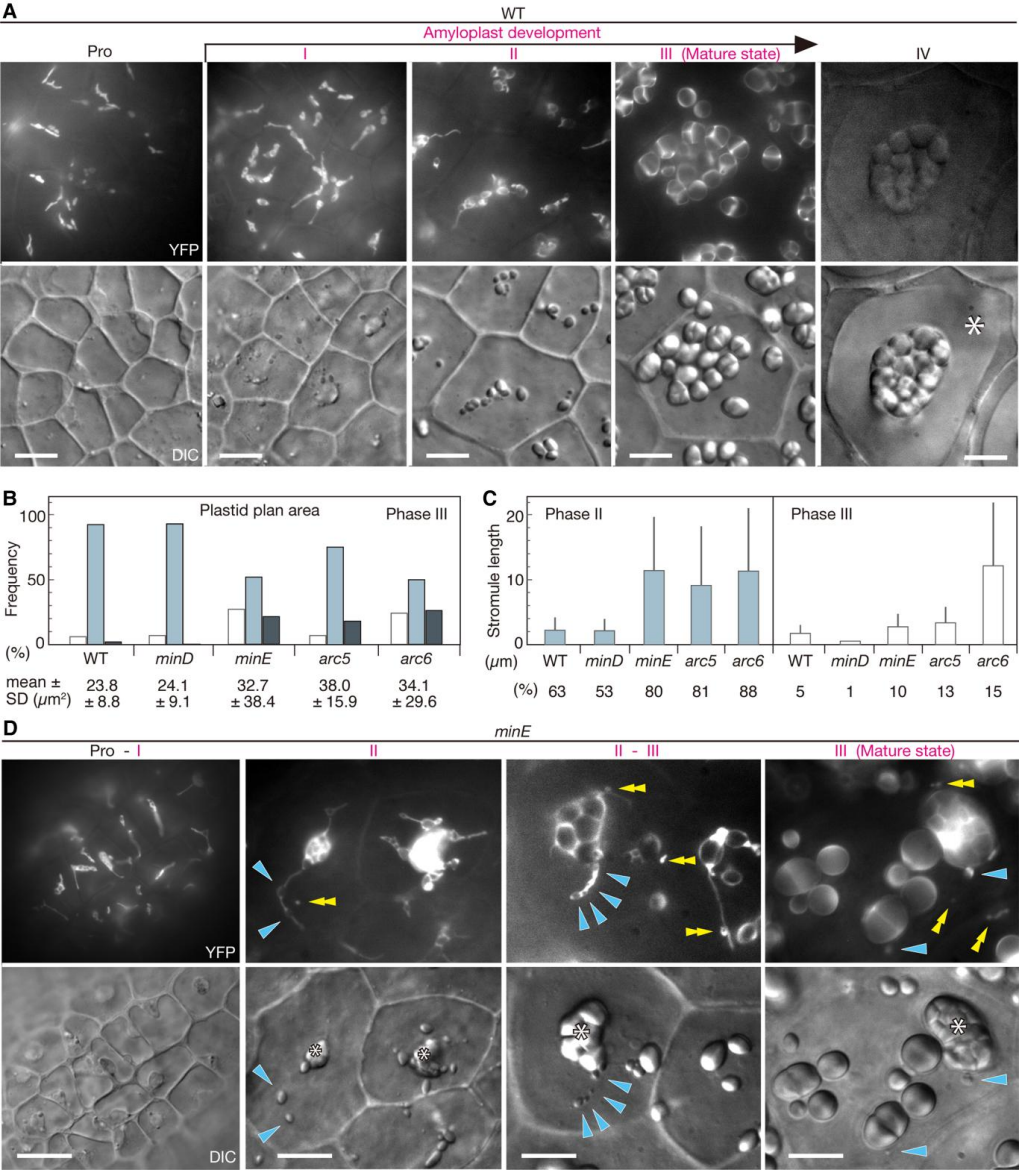
Developmental dynamics of amyloplast morphologies in WT and *minE* plants. Integument cells of wild-type (WT), *minE*, and other plastid division mutants expressing stroma-targeted fluorescent proteins were investigated by fluorescence microscopy. **A)** Amyloplast development in the WT. After their differentiation from proplastids (Pro) in meristematic cells, the amyloplasts undergo maturation and then start degenerating during seed coat formation (Phases I–IV; see main text). In the Phase IV images, an asterisk indicates a mucilage-storage region that restricts the cytoplasm towards the cell center and the periphery. Loss of YFP signal indicates cell death. **B)** Measurement of the plastid plan area in mature integument cells of the WT and four plastid division mutants (n = 100 plastids, from three independent plants). Bars represent the frequency (%) of plastids with a plan area of <10 *µ*m^2^ (left), 10–50 *µ*m^2^ (middle), or >50 *µ*m^2^ (right). Average values with SD are also shown. See also Supplementary Fig. S2. **C)** Stromule frequency and length in the plastids (Phases II and III) of WT and mutant plants. The frequency of stromules (%) (n = 150 plastids for Phase II and 100 plastids for Phase III) and their mean length (n = up to 50 plastids) were measured as described in MATERIALS AND METHODS. Error bars represent SD. See also Supplementary Fig. S5. **D)** Amyloplast development in the *minE* mutant. Asterisks indicate giant amyloplasts, and single and double arrowheads indicate the position of tiny starch grains and YFP-labeled plastids, respectively. Amyloplast development in *minD*, *arc5*, and *arc6* mutants is depicted in Supplementary Fig. S4. Scale bars: 10 *µ*m **(A, D)**.

To address whether or how amyloplast proliferation is differentially controlled from chloroplasts, we initially chose to characterize four Arabidopsis plastid division mutants (*minD*, *minE*, *arc5*, and *arc6*) and their corresponding wild-type accessions (Fig. 1, F to K). Amyloplasts at maturity (Phase III), when their proliferation activity is ceased (see Fig. 2), were comparatively examined by light microscopy, and the number of amyloplasts per cell was carefully counted (Fig. 1L and Supplementary Fig. S1). Wild-type cells contained round to ellipsoidal amyloplasts of relatively homogeneous size, although this homogeneity was less stringent than that of chloroplasts (Supplementary Figs. S2 and S3). Amyloplast number per cell (mean ± SD) varied among accessions (28.2 ± 8.0 in L*er*, 20.7 ± 5.6 in Ws, and 12.3 ± 2.8 in Col; Fig. 1L), and this distribution pattern might be related to that of leaf mesophyll chloroplasts (Marrison et al. 1999; Yoder et al. 2007). In *minD* (or *arc11*), a defective mutant in chloroplast division site positioning (Marrison et al. 1999; Fujiwara et al. 2004), amyloplast size was slightly heterogeneous, but amyloplast number was similar to the wild type (Fig. 1H). By contrast, *minE*, a chloroplast division mutant with one to several chloroplasts per leaf mesophyll cell (Fujiwara et al. 2008), showed a reduced number of heterogeneous amyloplasts, and the degree of amyloplast size heterogeneity varied between cells (Fig. 1I). *arc5*, which has a mutation in the gene encoding a dynamin-related protein (DRP5B) and has division-arrested chloroplasts (Pyke and Leech 1994; Gao et al. 2003), showed 50% fewer amyloplasts with modest enlargement and heterogenous phenotypes compared with the wild type (Fig. 1, J and L). Furthermore, *arc6*, which blocks chloroplast division initiation (Pyke et al. 1994; Vitha et al. 2003), exhibited a striking heterogeneity in amyloplast size, similar to *minE* (Fig. 1K). Overall, we found no conserved phenotypes with regard to proliferation and morphology between amyloplasts and chloroplasts in these mutants. This suggested that physiological importance of plastid division factors is distinct between amyloplasts and chloroplasts. This observation gave us a caution that the mechanisms of normal and aberrant amyloplast replication must be carefully examined and interpreted. Hereafter, we mainly focused on wild-type and *minE* mutant cells.

### Developmental processes of amyloplast formation in the wild-type and mutants

To understand how the amyloplasts of the wild-type and mutant were formed, we investigated their developmental morphologies in detail (Fig. 2 and Supplementary Fig. S4). Transgenes encoding stroma-targeted fluorescent proteins were introduced into these plants by crossing, and both the plastid shape and starch granule growth were monitored by fluorescence microscopy (see Materials and Methods). All transgenic plants grew and reproduced normally under standard Arabidopsis growth conditions. In addition, as shown by previous studies on plastid morphology (e.g. Holzinger et al. 2008; Schattat et al. 2012; Fujiwara et al. 2018), the morphology of amyloplasts was well conserved between the original and crossed plants (see Fig. 1, F and G, M–O).

The ovules of wild-type flowers at developmental stages 13–17 (Smyth et al. 1990) display the full sequence of amyloplast development (Fig. 2A) (Western et al. 2000; Windsor et al. 2000). The stroma-targeted fluorescent proteins colocalized with starch grains, reconfirming the identity of amyloplasts. Observations revealed that amyloplasts undergo dynamic morphological changes during differentiation, which could be divided into four phases (Fig. 2, A to C). Hereafter, starch-containing plastids are referred to as “amyloplasts”, while starchless plastids or plastids that cannot be strictly defined are simply referred to as “plastids”. During Phase I (amyloplast width = 1.5–2.5 *µ*m), when starch grains are barely detectable, plastids increase in size and adopt highly filamentous forms, leading to the proliferation of stromules. In Phase II (amyloplast width = 2.5–5.0 *µ*m), starch grains accumulate in the stroma, and the filamentous plastids become amorphous in shape, reducing the rate of stromule production. In Phase III (amyloplast width = 5.0–12.0 *µ*m), starch grains, which vary in number from one to several per amyloplast, occupy almost the entire amyloplast volume, and amyloplasts assume a spherical to ovoid shape and produce no or few stromules. In Phase IV, mature amyloplasts shift their position to the cell center because of cytoplasmic constriction (Western et al. 2000; Windsor et al. 2000), and this is followed by entry into starch degradation and cell death. Thus, the initiation of starch granules non-synchronously occurs during Phases I and II, but their subsequent growth seems relatively constant. We reason that this and proper amyloplast segregation result in moderately homogenous amyloplast populations in cells.

*minE* and other mutants were characterized as described above, with a focus on Phases I–III of amyloplast development. The sequence of amyloplast development in *minD* and *arc5* was similar to that in the wild type, although *arc5* showed extended amyloplast growth beyond the wild-type range (Supplementary Figs. S2). By contrast, both *minE* and *arc6* showed striking phenotypes (Fig. 2D and Supplementary Fig. S4). At the proplastid stage, selective enlargement of plastids was discernible. However, at subsequent stages, the stromules proliferated dramatically from large differentiating amyloplasts and then stabilized until Phase III, while the giant amyloplasts continued to grow. With respect to starch synthesis, granules arose in amyloplast bodies, predominantly in giant amyloplasts in correspond with amyloplast volumes, but could also occur in stromules. It can be a matter of argument whether the giant amyloplast-associated and starch-containing stromules originated from giant or small amyloplasts. Nevertheless, their developmental stability supports that stromules originated from giant amyloplasts. Concomitant to these symptoms, small globular to tubular plastids with or without starch grains frequently arose, so that giant amyloplasts and poorly developed amyloplasts and/or starchless plastids could coexist in cells.

General comparisons of plastid plan area and stromule length and frequency between wild-type and mutant plants are presented in Figs. 2, B and C and Supplementary Figs. S2 and S5. These results indicated that amyloplast heterogeneity in *minE* and *arc6* was caused by abnormal plastid replication; giant amyloplasts did not follow equal duplications, while new amyloplasts or starchless plastids were formed with only small sizes. This caused marked differences in the timing of starch granule initiation and growth (amyloplast differentiation) and their imbalanced partition (amyloplast segregation) within cells. In assessing the possible reasons for such abnormal amyloplast replications, the developmental stability of stromules could serve as a key element.

### Modes of amyloplast replication in wild-type and *minE* plants

The amyloplast phenotypes of *minE* and other mutants are hard to explain by the current models of chloroplast division. In particular, amyloplast multiplication in the mutant cells remained unblocked, raising questions on the amyloplast replication mechanisms with and without the gene functions. To elucidate the mechanisms of amyloplast replication, we monitored the behavior of FtsZ in wild-type and *minE* amyloplasts using the FtsZ1-GFP fusion protein as a probe (Fujiwara et al. 2008).

**Figure 3.**
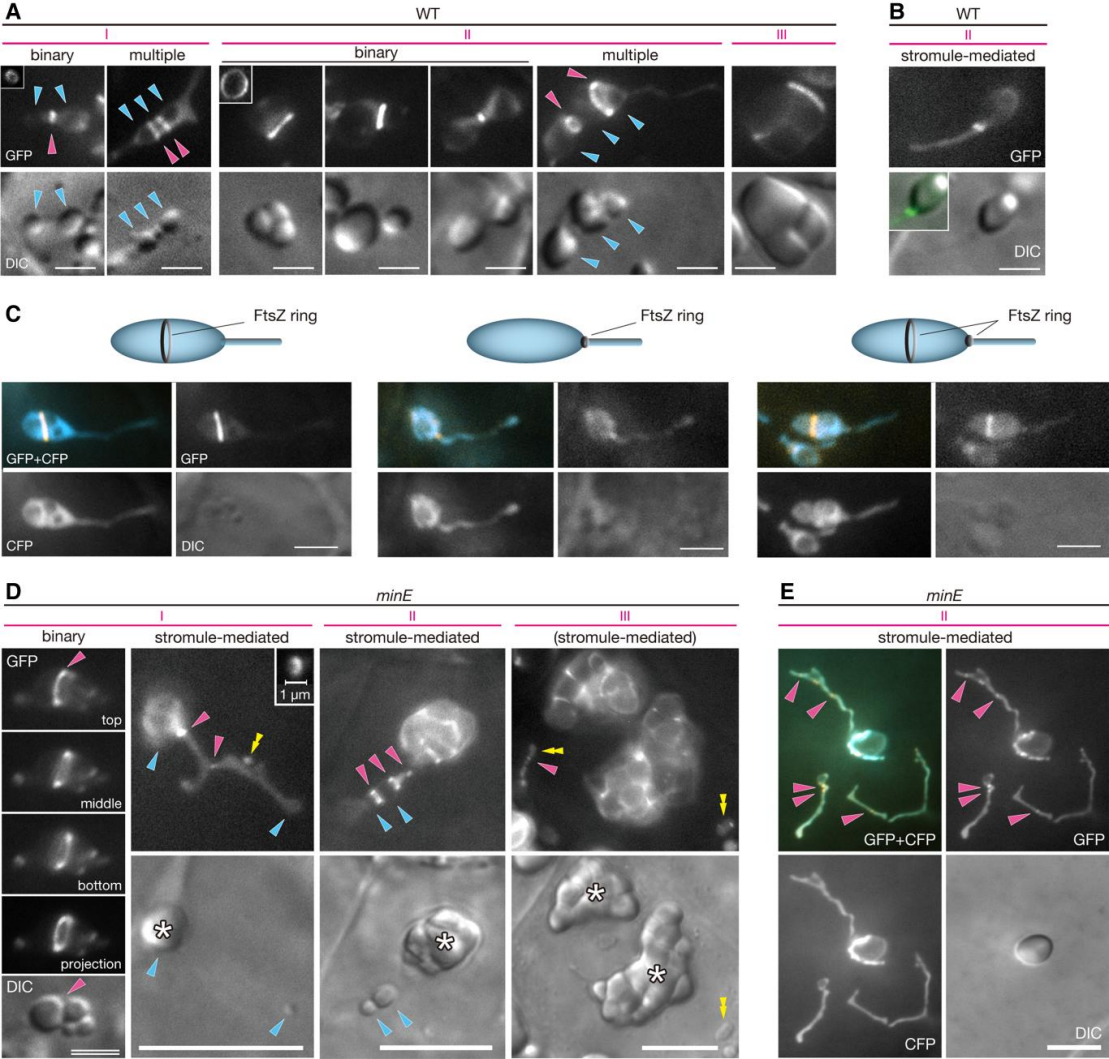
FtsZ ring formation during amyloplast replication in WT and *minE*. Integument cells of wild-type (WT) and *minE* plants expressing the FtsZ1-GFP probe with or without stroma-targeted CFP were microscopically characterized. **A)** WT amyloplasts that undergo binary and multiple fission. The insets indicate the configuration of the FtsZ ring. **B)** A WT amyloplast that undergoes stromule-mediated fission. The inset shows merged GFP (green) and differential interference contrast (DIC) images. **C)** FtsZ ring formation in stromule-extending amyloplasts. FtsZ ring was detected at either the plastid body or the stromule, or both. **D)***minE* amyloplasts that undergo binary and stromule-mediated fission. In the leftmost panel, three z-series images of FtsZ1-GFP (focusing on the top, middle, and bottom of a single amyloplast) and the projection image are shown. Asterisks and double arrowheads indicate giant amyloplasts and tiny fluorescent plastids, respectively. **E)***minE* amyloplasts that indicate stromule-mediated fission. In **(C and E)**, the merged images of CFP (cyan) and GFP (yellow) are pseudo-colored. In **(A, B, D, and E)**, the developmental stage and the replication mode of amyloplasts are indicated. The position of the following structures is also indicated: cyan arrowheads, starch grains; magenta arrowheads, putative FtsZ rings. See also Supplementary Figs. S7 and S8. Scale bars: 3 *µ*m **(A–C, D** [double line]**)** and 10 *µ*m **(D** [single lines]**)**.

The *FtsZ1* promoter-driven *FtsZ1-GFP* fusion was expressed in integument cells (Fig. 3A and Supplementary Figs. S6 and S7). Fluorescence microscopy using FtsZ1-GFP and FtsZ1-GFP-ox plants (Fujiwara et al. 2009), as well as western blot analysis using anti-GFP and anti-FtsZ1 antibodies (Vitha et al. 2001), confirmed that the FtsZ1-GFP protein was localized to amyloplasts and the fluorescent signal was basically derived from the intact fusion protein in the cells (Supplementary Fig. S6, A to C). The number and morphology of amyloplasts in the *FtsZ1-GFP* line were also similar to those of non-transgenic plants (Supplementary Fig. S6D).

During Phases I and II, wild-type cells showed the formation of one to several (up to four) FtsZ rings encircling the amyloplast body along its length (Fig. 3A and Supplementary Fig. S7). Dot and short filament forms were occasionally detected within faint stromal diffusions, which enabled us to monitor organelle shapes and FtsZ configurations simultaneously. The localization of FtsZ rings at central or non-central amyloplast sites and at constriction and non-constriction sites, together with the non-synchronous progression of constrictions at multiple-arrayed amyloplasts, suggested that amyloplasts follow binary and multiple fission modes using FtsZ rings. Unexpectedly, however, we also found minor FtsZ ring formation at stromules (Fig. 3, B and C and Supplementary Fig. S8). A single FtsZ ring tended to stay and constrict the stromule at the neck region, which connects to the amyloplast body, whereas faint diffusion signals were distributed along the length of the stromule, irrespective of FtsZ ring formation. Co-expression of the stroma-targeted CFP and FtsZ1-GFP confirmed that stromule-extending amyloplasts contained no or 1–2 FtsZ rings at the amyloplast body and/or the stromule (Fig. 3C and Supplementary Fig. S8). The physiological significance of this stromule-localized FtsZ ring could not be directly determined, but the presence of stromules with a deep constriction (Supplementary Fig. S8C) may point toward successful stromule fission events. The frequency of FtsZ ring formation at stromules was estimated by observing FtsZ ring formation in over 200 differentiating Phase I amyloplasts (see Supplementary Fig. S9). As a result, over 15% of FtsZ rings (38/212 FtsZ rings, n = 2 plants) were detected. Together, our observations indicate the involvement of three types of amyloplast replication modes, binary fission, multiple fission, and stromule-mediated replication, during the proliferation of wild-type amyloplasts.

The *minE* mutant showed additional novel features of FtsZ1 patterning (Fig. 3D). Rather than division rings, the body parts of giant amyloplasts contained short filaments or dots with faint diffusions. This is consistent with the phenotypes of leaf mesophyll chloroplasts and the morphology of amyloplasts (Fig. 2). Despite this, FtsZ rings were formed in poorly developed amyloplasts or starchless plastids with ∼2 *µ*m diameter. Moreover, FtsZ rings were also found in stromules in *minE* and at a higher frequency than in the wild type. These FtsZ rings of *minE* appeared during Phases I–III and varied from one (or zero) to several per amyloplast, merging with the organelle periphery. These results imply that the formation of FtsZ rings is dependent on amyloplast (plastid) size in *minE*. To further address the role of these FtsZ rings in plastid constriction in *minE*, we introduced another reporter gene, *CFP*, into FtsZ1-GFP-expressing *minE* plants to label the stroma (Fig. 3E and Supplementary Fig. S8, F and G). Dual fluorescence signals revealed the localization of FtsZ rings at narrow sites of small-sized amyloplasts (plastids) and stromules, although many of these rings appeared as extremely short lines or dots, like those observed at the final stages of chloroplast division (Vitha et al. 2001; Kuroiwa et al. 2002). In *minE* stromules, we observed both constriction sites and non-constriction sites associated with putative FtsZ rings, as well as narrow sites not associated with FtsZ rings (Fig. 3E and Supplementary Fig. S8, F and G). We presumed that the FtsZ rings in *minE* stromules may facilitate the constriction process, although the stromule may undergo narrowing without an aid of FtsZ1. Together, these data indicate that three modes of amyloplast replication operate in *minE* as well as wild-type amyloplasts. Nevertheless, *minE* mutant cells showed a shift towards stromule-mediated replication, and this mode of replication had moderate impairments in the FtsZ ring-mediated envelope fission. It is likely that the greater extent of stromule development and the reduced, but remaining, activity of FtsZ ring assembly are tightly linked to these effects.

### Live imaging reveals enhanced stability of stromules in *minE*

Our results provide a plausible explanation for how the stromules supply new daughter plastids for amyloplast proliferation in wild-type and *minE* Arabidopsis plants. Yet, stromules generally move bidirectionally within minutes or even seconds. It must be equally questioned whether the wild-type or *minE* stromules of developing amyloplasts continually exist or can retract soon after formation. Extended time-lapse microscopy of fluorescence-labeled wild-type and *minE* stromules was developed and performed.

**Figure 4.**
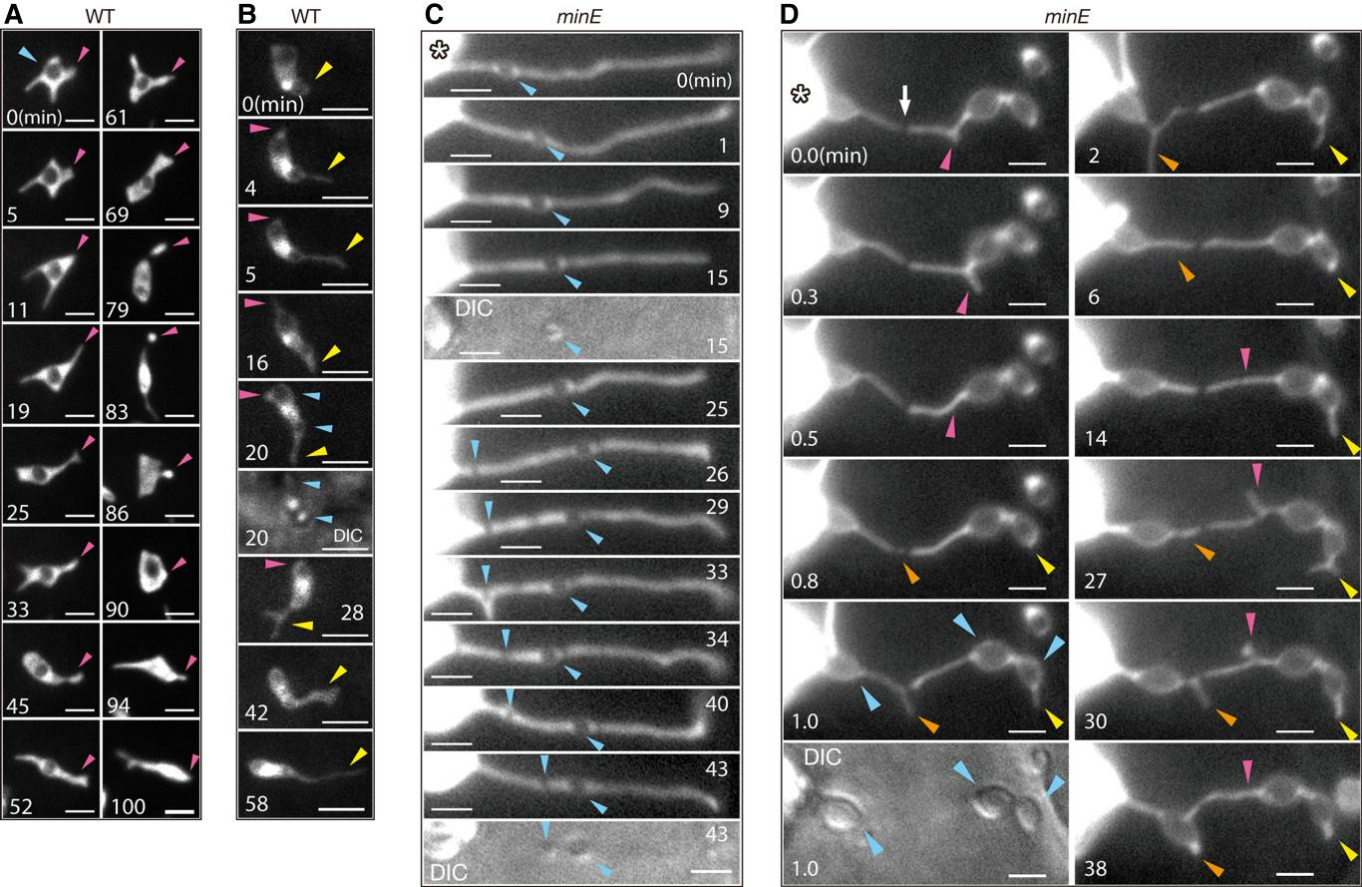
Dynamic behavior of amyloplasts or stromules in WT and *minE* cells. Time-lapse fluorescence microscopy was performed on amyloplasts from late Phase I to early Phase II. **A, B)** Wild-type (WT) amyloplasts that exhibit morphological alterations including the repetitive movement of stromule extension and retraction **(B)**. **C, D)***minE* amyloplasts that exhibit long stromules with relatively small **(C)** and large **(D)** starch grains therein. Images were taken at 1 min or shorter intervals, and representative images are shown. Cyan arrowheads indicate the position of starch grains, while magenta, yellow, and orange arrowheads follow the fate of stromules. Asterisks indicate giant amyloplasts. See also Supplementary Video S1. Scale bars: 2 *µ*m.

Selected time-lapse images of wild-type amyloplasts are presented in Fig. 4, A and B. Wild-type stromules, which were ∼3 in number and ∼9 *µ*m in length per amyloplast, had varied but relatively short-lives on the whole. Developing amyloplasts showed fluid envelope membranes and active envelope remodeling and motility. The repetitive extension and retraction movements of stromules were evident from the changing amyloplast shapes, and may explain that, during stromule retraction towards the amyloplast body, an FtsZ ring tends to shift to the stromule neck (Supplementary Fig. S8C). Thus, the long life-span or stability of stromules permitted the formation of FtsZ ring therein. The motility of FtsZ structures in the stromule (Supplementary Fig. S8) may indicate a way for the determination of the FtsZ ring assembly within amyloplasts.

In *minE*, stromules were associated with giant amyloplasts in a stable manner. Their behavior (Supplementary Video S1 and Fig. 4) indicated that these stromules were derived from giant amyloplasts rather than that the stromules were associated with the edges of the giant amyloplasts. Notably, stromule movements at local regions, such as branching points and apical growth regions, were almost similar to those in the wild type (Fig. 4, C and D). This may possibly explain our occasional detection of FtsZ1 signals at the edges of stromule-like protrusions, since the extreme stromule retractions would force the transposition of the contractile FtsZ ring to the tip (Supplementary S8F). It is considered that the basic mechanisms governing stromule movements are conserved in *minE*, and the phenotype of giant amyloplast–stromule associations in *minE* is a consequence of the increased elongation and stability of stromules. While analyzing the temporal behavior of *minE* stromules, we detected starch movements within stromules (Fig. 4C). This to-and-fro movement was reminiscent of previously observed GFP behavior (Köhler et al. 2000), and perhaps indicates one of the routes of starch grain formation and growth in stromules. These results address the similarities and differences in stromules between wild-type and *minE* amyloplasts.

### Distribution of nucleoids in developing amyloplasts and stromules of *minE*

In *E.coli*, the *minB* operon encodes three cell division-regulator genes, *minC*, *minD* and *minE*, which coordinately define the midcell division site (de Boer et al. 1989). Mutations in these genes could cause asymmetric cell division, leading to heterologous cell populations, including the production of anucleate mini cells. In an attempt to address whether the Arabidopsis *minE* mutation can affect nucleoid distribution in small amyloplasts or starchless plastids, we performed DNA staining of integument cells with the DNA-binding dye, SYBR Green (Supplementary Fig. S10).

Developing ovules (Phase II) excised from stromal CFP-expressing wild-type and *minE* plants were fixed with 4% (w/v) paraformaldehyde and stained with a 1/1,000-diluted SYBR Green solution. Fluorescence microscopy detected nucleoids in the wild-type amyloplasts (Supplementary Fig. S10A). Integument cells contained numerous mitochondria, whose nucleoids exhibited clear dot or rod-like signals, whereas plastid nucleoids tended to show relatively weak fluorescence with dotted, elongated, or even blurred signal patterns. These variable nucleoid morphologies could be attributed to the presence of starch grains in the stroma. On the other hand, we found several unique nucleoid distributions in *minE*. First, some small amyloplasts and starchless plastids had nucleoids, whereas others had not (Supplementary Fig. S10B and C). Moreover, nucleoids were occasionally present in stromules of *minE* amyloplasts (Supplementary Fig. S10D), in contrast to the preferential location of wild-type plastid nucleoids at the plastid body (Newell et al. 2012). Although more experiments are needed to establish the behavior of plastid nucleoids during amyloplast development in wild-type and *minE* integument cells, these observations suggest that the *minE* mutation perturbs not only the proliferation and morphology of amyloplasts but also the distribution of their nucleoids in integument cells.

### Amyloplast development and replication in *ftsZ* null mutant

To demonstrate the role of FtsZ in amyloplast replication, we analyzed the Arabidopsis *ftsZ1-1 ftsZ2-1 ftsZ2-2* triple (*ftsZ* null) mutant (Schmitz et al. 2009; hereafter termed as *ftsZ*). A transgenic *ftsZ* line expressing the stroma-targeted *YFP* gene was generated by *Agrobacterium*-mediated transformation. The progeny plants were analyzed by fluorescence microscopy, as described above for wild-type and *minE* plants.

**Figure 5.**
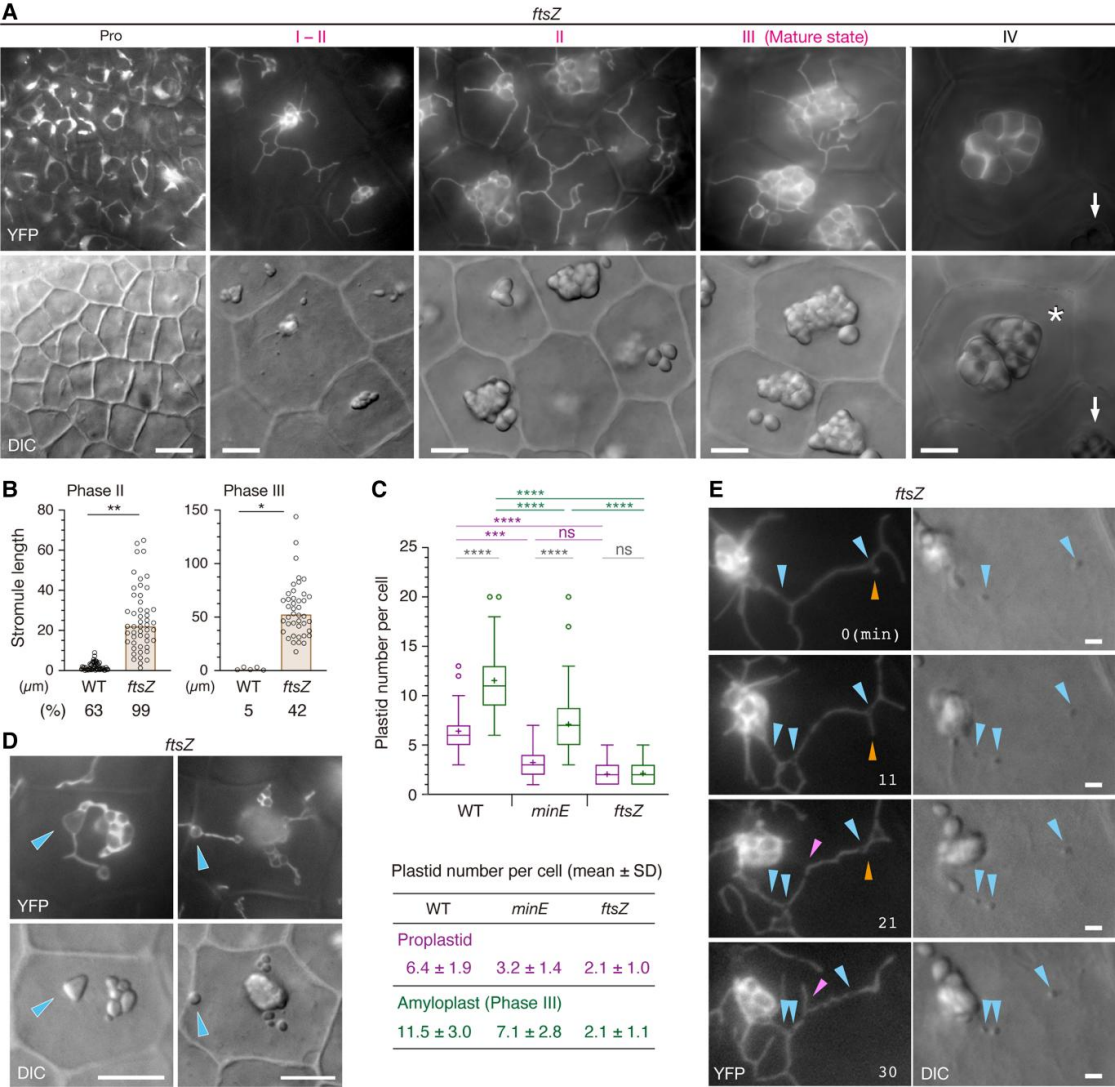
Characterization of amyloplast morphologies in the integument cells of *ftsZ* expressing stroma-targeted YFP. **A)** Differentiation and maturation of amyloplasts. Amyloplasts differentiate from proplastids (Pro) and then mature over four developmental stages (Phases I–IV; see Fig. 2A). Asterisk indicates a mucilage-storage region and arrows indicate diminishing YFP signals. **B)** Measurement of stromule frequency and length in WT and *ftsZ* plastids (Phases II and III). The frequency of stromules (%) (n = 150 plastids for Phase II and 100 plastids for Phase III) and their mean length (reproduced from Fig. 2C for wild-type (WT)) were measured using the images of YFP-expressing WT and *ftsZ* plants, as described in MATERIALS AND METHODS. A nested *t* test was used to determine significant differences (* *P* < 0.05; ** *P* < 0.01) between WT and *ftsZ* in both phases. **C)** Boxplots showing the number of plastids in cells. YFP-expressing WT, *minE*, and *ftsZ* plants were used to count the number of plastids per cell (n = 100 cells, from three independent plants, except for *minE* proplastids, which were derived from four plants). Elements of the boxplot are described in MATERIALS AND METHODS. Average values with SD are also shown. A nested one-way ANOVA with Tukey's multiple comparison test was performed to determine the differences between the samples using data from three plants (*** *P* < 0.001; **** *P* < 0.0001; ns, not significant). See also Supplementary Fig. S12. **D)** Starch grains in stromules of *ftsZ* amyloplasts (Phase II). **E)** Time-lapse fluorescence microscopy of *ftsZ* amyloplasts (Phases I and II) in the same sample as that used in Fig. 5A. Representative images are shown. In **(D)** and **(E)**, cyan arrowheads indicate the position of starch grains, while magenta and orange arrowheads follow the fate of stromules. See also Supplementary Figs. S11 and Supplementary Videos S2 and S3. Scale bars: 10 *µ*m **(A, D, E)**.

The *ftsZ* mutant is fertile and can develop intact ovules, including integuments (Supplementary Fig. S11). Starch grains were formed in *ftsZ* during the development of integument cells; however, amyloplast morphology was more severely impacted in *ftsZ* compared with *minE* (Fig. 5A). Proplastids, which varied in number from one to several per cell (see below), were noticeably elongated in *ftsZ* and showed amorphous shapes. Starch grains occurred preferentially in plastid bodies and rarely in stromules. Both the number and length of stromules were considerably increased throughout Phases I–III. Because of the three-dimensional growth and high mobility of stromules, total stromule length per amyloplast could be measured only in the top plane of cells, yet an extreme level of stromule activation was still evident (Fig. 5B). The mobility of stromules in cells and that of starch grains in stromules were similar in *ftsZ* and *minE* (Fig. 5, D and E). Nevertheless, unlike *minE*, the *ftsZ* mutant critically lacked small plastids without starch grains in integument cells (Figs. 2D and 5). Despite their hyper-activation, stromules seemed to be incorporated into giant amyloplasts, irrespective of the existence of starch grains.

To quantitatively assess the occurrence or absence of stromule fissions in *ftsZ*, we counted the number of proplastids and amyloplasts (Phase III) per integument cell using *YFP*-expressing wild-type, *minE*, and *ftsZ* plants (Fig. 5C). In wild-type plants, 5–8 proplastids increased to 9–15 amyloplasts per cell, whereas in *minE*, 2–5 proplastids proliferated to 4–10 amyloplasts. By contrast, in *ftsZ*, the plastid number per integument cell remained within a range of 1–3 throughout the integument amyloplast development. These results clearly supported that without FtsZ, amyloplast replication is blocked in integument cells. Thus, FtsZ is indispensable not only for the division of wild-type amyloplasts but also for the fission of poorly developed amyloplasts and stromules in *minE* (Fig. 3).

### Formation of the MinE ring at the putative division site of amyloplasts

Finally, we examined the expression and localization of MinE in integument cells. In a previous study, immunofluorescence microscopy revealed that MinE exists at the chloroplast division site and at dispersed punctate structures on the stromal side of the inner envelope upon the initiation of chloroplast division (Miyagishima et al. 2011). In this study, we investigated MinE activity in *minE* plants expressing a *MinE-YFP* fusion under the control of the *MinE* promoter (i.e. *MinE*p::*MinE-YFP*/*minE* complementation line) (Fujiwara et al. 2015) using fluorescence microscopy.

**Figure 6.**
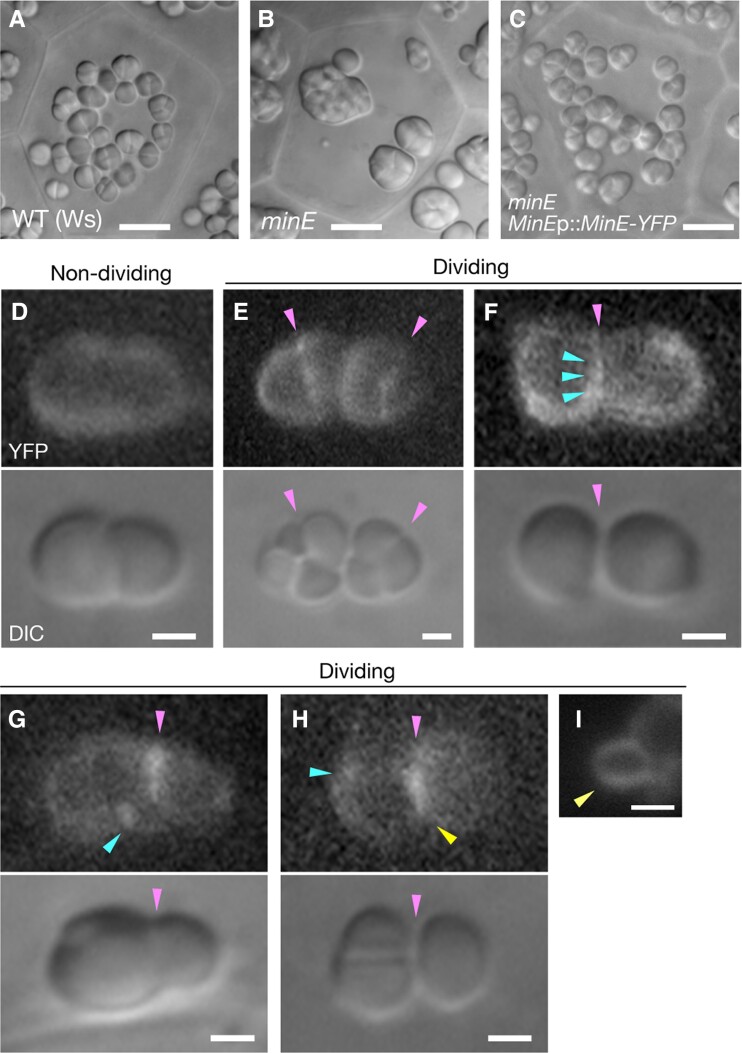
Expression and localization of the MinE-YFP fusion protein in the integument cells of transgenic Arabidopsis plants. **A–C)** Complementation of the abnormal amyloplast morphologies of *minE* by the expression of *MinE-YFP* fusion under the control of the *MinE* promoter (*MinE*p::*MinE-YFP*). **A)** WT control. **B)***minE*. **C)** Transgenic *minE* plant with *MinE*p::*MinE-YFP*. **D–I)** Detection of MinE-YFP signals in amyloplasts. A non-dividing amyloplast **D)** and putative dividing amyloplasts with MinE-YFP signals in the form of discontinuous **(E and F)** or continuous **(G–I)** filaments were observed. In **(I)**, a ring-like configuration of MinE-YFP is shown. Magenta arrowheads indicate the ring-like structure of MinE at the putative amyloplast division site(s). Cyan and yellow arrowheads indicate punctate and filamentous forms of MinE, respectively. Scale bars: 10 *µ*m **(A–C)**)and 2 *µ*m **(D–I)**.

The expression of *MinE*p::*MinE-YFP* complemented the abnormal morphology of amyloplasts in *minE* integuments (Fig. 6, A to C). Similar to the results of FtsZ ring detection (Fig. 3 and Supplementary Fig. S7), amyloplast division sites could not be easily predicted using bright-field images only; however, diffused fluorescence signals were observed within the stroma of putative non-dividing amyloplasts (Phase II), except in the starch grain space (Fig. 6D). This diffusion was not necessarily uniform, as the local accumulation of fluorescence signals was observed. On the other hand, in putative dividing amyloplasts, MinE-YFP signals were detected at non-constriction (Fig. 6E) and constriction (Fig. 6, F to H) sites of amyloplasts in the form of discontinuous (Fig. 6, E and F) or continuous (Fig. 6, G and H) filaments. These structures can form a ring-like structure at constriction sites (Fig. 6I). These data indicated that MinE is expressed in integument cells to regulate the assembly and control of the amyloplast division apparatus, as in chloroplasts.

## Discussion

Our results provide a basis for analyzing and interpreting the regulatory mechanisms of amyloplast replication in plant cells. Starch metabolism in amyloplasts or chloroplasts is essential for plants, and dissecting the role of amyloplasts in determining organelle size and number is important in plant science (e.g. Yamamoto et al. 2002; Crumpton-Taylor et al. 2012). Since a collection of non-photosynthetic plastids arose in plant cells after the endosymbiotic acquisition of chloroplasts, and thus represent later versions of plastids (Pyke 1999), a significant part of the regulatory mechanisms in non-photosynthetic plastids, including amyloplasts, may have developed and been adapted from basic chloroplast mechanisms over time. Whether the mechanism of division is universal across all plastid types has been an important topic of research in plastid biology (Pyke 1999; Okazaki et al. 2009). While the FtsZ ring has proven to be a general component of the envelope-fission apparatus of both chloroplasts (Osteryoung and McAndrew 2001) and amyloplasts (this study), our results revealed that the regulatory basis of amyloplast replication is complex and differs from that of chloroplast replication. Thus, the results of this study may facilitate further studies in related fields.

As a methodological breakthrough, the Arabidopsis ovule integument (Schneitz et al. 1995; Western et al. 2000; Windsor et al. 2000; Young et al. 2008) has emerged as a live system for studying amyloplast biogenesis and replication. Thus far, amyloplast research has been conducted using tubers or endosperms of agriculturally important crops, the root cap columella of various plants, or the suspension culture of tobacco (*Nicotiana tabacum*) BY-2 cell line (Kirk and Tilney-Bassett 1978; Thomson and Whatley 1980; Sakai et al. 1992). While these systems have specific merits, a platform allowing both genetic and molecular analyses of amyloplast development, in combination with omics-based approaches, molecule-, organelle-, and cell-specific markers, and live microscopy, was needed. The integument system used in this study satisfies many of these criteria and may open up avenues for research on amyloplasts.

### Essential differences between amyloplast and chloroplast replication

The formation of one or a few giant plastids per cell by blocking the plastid division apparatus has been accepted as a standard for chloroplast division in photosynthetic cells (Osteryoung et al. 1998; Strepp et al. 1998; Vitha et al. 2003; Schmitz et al. 2009). However, this paradigm does not apply to several kinds of plastids (Forth and Pyke 2006; Holzinger et al. 2008; Chen et al. 2009; Kojo et al. 2009; Swid et al 2018). To explore a paradigm for amyloplast replication, we employed Arabidopsis mutants, mainly *minE*, in which chloroplast FtsZ ring assembly is impaired, like that in *arc6* (Vitha et al. 2003; Fujiwara et al. 2008). In the *minE* mutant, integument cells showed amyloplast heterogeneity, indicative of abnormal division sites, based on the results of chloroplast experiments. This led us to question how *minE* amyloplasts could efficiently proliferate in cells. Results of detailed microscopic analyses of plastids of *suffulta* (*su*) (Forth and Pyke 2006) and *arc6* (Holzinger et al. 2008; Chen et al. 2009; Fujiwara et al. 2018) mutants were crucial to our current study. In both *su* and *arc6*, like *minE*, 1–3 giant chloroplasts are found per mesophyll cell, whereas wild-type-like populations of chromoplasts or numerous, heterogeneously sized plastids are generated in the ripening fruit or several epidermal cells, respectively. Giant chloroplasts in *su* appeared to degenerate during transdifferentiation into chromoplasts, which involved the budding and fragmentation of plastids or stromules. Our current analysis of *minE* revealed, at the molecular level, how plastids (amyloplasts) can proliferate even in severe chloroplast-division defective mutants. The principle of this outcome, i.e. the size-dependency of plastids for replication, may support and complement the interpretations of the two mutant phenotypes: (1) the generation of poorly developed plastids with or without starch grains through this mechanism causes differential amyloplast development within cells; (2) unusual FtsZ ring positioning in *minE* stromules contributes to amyloplast heterogeneity; and (3) stromule fissions in *minE* result in globular and filamentous plastids, whereas FtsZ-mediated fission is the major route of plastid proliferation. This mechanism of plastid proliferation might be applicable to *su* beyond the developmental status of plastids. Given the shared plastid multiplication mechanisms in non-photosynthetic tissues of plastid morphogenesis mutants/transgenics (Forth and Pyke 2006; Holzinger et al. 2008; Chen et al. 2009; Wang et al. 2013) and our previous study on *minE* and *arc6* (Kojo et al. 2009; Fujiwara et al. 2015; Fujiwara et al. 2018), the present study provides crucial insights into plastid biology. We hypothesize that the replication of plastids of optimal size is negatively correlated with the severity of defects in the division apparatus, while this compensation system requires the involvement of FtsZ (Figs. 3 and 5 and Supplementary Fig. S8).

A follow-up question from this is the amyloplast replication mechanisms in wild-type cells. Investigation of the spatiotemporal behavior of FtsZ1 in organelles revealed essential similarities and differences between amyloplast and chloroplast replication in wild-type plants. First, the FtsZ ring serves as a common player in plastid envelope fission in both amyloplasts and chloroplasts. FtsZ ring appears from non-constricting sites to deep-constricting sites of amyloplasts, like that in chloroplasts. Second, the spatial control of FtsZ ring formation differs between amyloplasts and chloroplasts. Notably, the division of wild-type amyloplasts is similar to that of *minD* chloroplasts (symmetric, asymmetric, and multiple) (Fujiwara et al. 2008). Hence, what is considered unusual in chloroplasts seems to be the default situation in amyloplasts. Third, FtsZ ring placement at the stromule neck is a unique characteristic of the amyloplast proliferation process (Fig. 3B and Supplementary Fig. S8C). While rare, this event does occur; however, the detailed mechanism involved in achieving this FtsZ ring placement remains to be fully resolved. Since starch grains occasionally appear in stromules and prevent the discrimination among amyloplast replication modes, the significance of the role of stromules in plastid replication might be greater than that speculated in the current analysis. A question that then arises concerns the degree of complexity of amyloplast replication. It is noteworthy that the transformation of organelles, which enables the transposition of the FtsZ ring, is specific to amyloplasts, whereas a single FtsZ ring is stably formed at the equator of chloroplasts. However, the basis of the formation of multiple FtsZ rings per amyloplast remains unclear. One possible explanation is that the dominance of MinE over MinD (Fujiwara et al. 2008) controls the FtsZ states in integument cells.

The similarities and differences in amyloplast replication between wild-type and *minE* plants indicate that complex, but basic (wild-type), modes of amyloplast replication are flexibly reproduced in *minE*, involving the operation of the plastid size-dependent FtsZ ring assembly and function (Supplementary Fig. S8). Furthermore, FtsZ activities in plastid-envelope fission systems are crucially supported by the structural plasticity of plastids and stromules. A significant question remains as to how the perturbations in plastid division enhance stromule production. It is likely that extensive membrane production by plastids during growth contributes to stromule formation (Mathur 2021). Membrane extensions are a common feature of organelles such as plastids, mitochondria, and peroxisomes, and facilitate the interactions of these organelles with the endoplasmic reticulum (ER) (Mathur 2012; Mathur 2020; Mathur et al. 2023). Our recent study suggested a pivotal role of ER in stromule formation; the knockout (KO) mutation of *TRIGALACTOSYLDIACYLGLYCEROL5* (*TGD5*), which encodes a subunit of the plastid envelope-localized TGD complex involved in ER-to-plastid lipid transfer, leads to excessive stromule formation in various Arabidopsis tissues (Itoh et al. 2021). Thus, it is possible that ER–plastid interactions play some roles in amyloplast proliferation in wild-type as well as *minE* integument cells.

### Roles of stromules in plastid replication

When considering the unique features of amyloplast replication control, the stromule is a key factor. Stromules, emanating from the surface of plastids with 0.2–0.8 *µ*m diameter, have been implicated in plastid metabolism, homeostasis, and plant defense (reviewed in Hanson and Hines 2018). Our current data indicate that stromules can act as donors of new plastids via either an inherent (in wild-type) or an extensively acquired (in *minE*) mechanism. In comparison with conventional chloroplast division, stromule fission has two merits for plant cells: (1) facilitation of FtsZ ring assembly with the reduced scale of FtsZ polymerization and bundling; and (2) reduced requirement for energy and materials to complete the fission process. Overall, stromules provide a microspace for efficient FtsZ ring assembly, facilitating minimal envelope-fission processes, when their structural stability is achieved. In other words, the transformation of plastids, rarely achieved in chloroplasts, unexpectedly occurs upstream of FtsZ ring assembly.

The analysis of *ftsZ* integument cells showed that stromule fission within cells is a positively regulated process rather than an auto-collapsing one. Our data show that FtsZ is essential for stromule fission in integument cells if the long-lived stromule formation is provided, despite the impaired activity of the plastid division apparatus. It is possible that the FtsZ ring in *minE* stromules not only prevents the cancellation of the contraction state but also enables a rare event of stromule fission at its site.

### Future perspectives

The mechanism of amyloplast replication in plants and crops has long been a topic of research (Buttrose 1960; Hoshikawa 1968; Rose and Possingham 1976; Parker 1985; Mingo-Castel et al. 1991; Duckett and Ligrone 1993; Kawasaki et al. 1998; Langeveld et al. 2000; Bechtel and Wilson 2003; de Pater et al. 2006; Sagisaka 2008; Yun and Kawagoe 2009; Wei et al. 2010; Yun and Kawagoe 2010; Kamau et al. 2015; Pfotenhauer et al. 2023; Esch et al. 2023). Early electron microscopy studies indicated that amyloplasts replicate by budding (Buttrose 1960), multiple fission (Hoshikawa 1968), or binary fission (Rose and Possingham 1976; Mingo-Castel et al. 1991), while other studies proposed the role of organelle segmentation and fusion in controlling organelle number. It has now become evident that unequivocal identification of the replication mode is difficult based on the static images of amyloplasts only. To identify the mode of amyloplast replication, it would be essential to consider the ever-changing shape of developing amyloplasts (Fig. 4, and Supplementary Video S1), with information on the envelope-fission machinery. Importantly, the formation and growth of starch grains and the location of FtsZ ring assembly in plastids could affect the replication mode of amyloplasts (Fig. 3 and Supplementary Fig. S8). For example, the interconnected appearance of amyloplasts could be interpreted as single amyloplasts which are either replicating as in Arabidopsis *minE* (Fig. 3) or integrating as in Arabidopsis *ftsZ* (Fig. 5). It might be also considered that both occur simultaneously in each cell. Therefore, the observations made in this study might offer clues to understand the cause-and-effect relationships underlying amyloplast proliferation in various plants and could expand the possibilities for interpretating the hitherto accumulated data.

Basic information related to amyloplast replication obtained in this study highlights many unknowns, including the uncharacterized genes that control amyloplast number by amyloplast division, unknown plastid division genes with universal or amyloplast-specific expression, and other genes that may regulate the plastid division genes or proteins during the plant life cycle. Additional studies are needed to address these unknowns.

A working model of amyloplast replication in Arabidopsis integument cells is shown in Supplementary Fig. S13. This study may serve as a reference for the investigation of amyloplast replication in other Arabidopsis tissues and other plant species including commercial crops. The control of amyloplast proliferation in crops may be different from that in Arabidopsis because of evolution and domestication. Extension of our current methodologies to the study of storage amyloplasts in crop plants may enhance our understanding of starch synthesis in staple food crops such as rice and wheat.

## Materials and methods

### Plant materials and growth conditions

Arabidopsis (*Arabidopsis thaliana* (L.) Heynh.) accessions Wassilewskija (Ws), Landsberg *erecta* (L*er*), and Columbia (Col) were used as the wild type in this study. A T-DNA insertion mutant of *MinE*, Flag_056G07 (DLFTV7T3, Ws background), was obtained from Institut National de la Recherche Agronomique (INRA, France) (Samson et al. 2002). Recessive *arc* mutants, *arc5* (*arc5-1*, L*er* background; Pyke and Leech 1994), *arc6* (*arc6-1*, Ws background; *arc6-2* and *arc6-3*, L*er* background; Pyke et al. 1994), and *arc11*/*minD* (*arc11-1*, L*er* background; Marrison et al. 1999), were obtained from the Arabidopsis Biological Resource Center (Ohio State University, Columbus, OH, USA). The *ftsZ* triple KO mutant (Col background; Schmitz et al. 2009) was generously provided by Prof. Katherine W. Osteryoung (Michigan State University, MI, USA).

Transgenic lines expressing the transit peptide (TP)-fused *YFP* (TP derived from the N-terminal 90 aa of AtFtsZ1-1; Chen et al. 2009; Fujiwara et al. 2018), *GFP* (TP derived from the N-terminal 55 aa of RBCS1A; Niwa et al. 1999), and *CFP* (TP derived from the N-terminal 90 aa of AtFtsZ1-1; Fujiwara et al. 2015) genes, as well as those expressing *FtsZ1-GFP* (*FtsZ1-GFP* and *FtsZ1-GFP*-ox lines; Col background; Fujiwara et al. 2009) and *MinE-YFP* (*minE* background; Fujiwara et al. 2015), have been described previously. Fluorescent mutant lines derived from *arc5* × TP-YFP, *arc5* × TP-GFP, *arc6* (*arc6-3*) × TP-YFP, *arc6* (*arc6-3*) × TP-GFP, *minD* × TP-GFP, *minE* × TP-YFP, *minE* × TP-CFP, TP-CFP × FtsZ1-GFP, and *minE* × (TP-CFP × FtsZ1-GFP) crosses were either described previously (Fujiwara et al. 2015; Fujiwara et al. 2017; Fujiwara et al. 2018) or generated in this study. To confirm the data of fluorescent mutant lines, the *arc5* × TP-CFP, *arc6* × TP-CFP, *minD* × TP-CFP, and *minE* × TP-CFP (Kojo et al. 2009; Fujiwara et al. 2015; Fujiwara et al. 2017; this study) lines were arbitrarily used. The *ftsZ* triple KO mutant was transformed with *Agrobacterium tumefaciens* carrying the *TP-YFP* fusion (see below). The T_3_–T_5_ or F_3_–F_4_ progenies were used for the microscopic characterization of amyloplast phenotypes. Note that the expression of plastid-targeted fluorescent proteins had no harmful effects on the development and proliferation of amyloplasts. The data of *TP-YFP*-expressing wild-type and *minE* plants were mainly shown in this study because of low background signals during fluorescence microscopy.

Seeds were surface-sterilized and sown on 0.7% (w/v) agar-containing Murashige-Skoog (MS) medium (Murashige and Skoog 1962), as described previously (Fujiwara et al. 2008). After germination, seedlings were transferred to soil and grown under a long-day photoperiod (16 h light/8 h dark) with white light illumination.

### Fluorescence microscopy

Intact ovules extracted from pistils or green siliques collected from 5- to 8-week-old plants were whole-mounted on glass slides and covered with glass coverslips. The samples were observed with an inverted microscope (model IX70 or IX71; Olympus, Tokyo, Japan), equipped with a mercury lamp and a digital camera (model ORCA-ER or ORCA-flash 2.8, Hamamatsu Photonics, Hamamatsu, Japan), using 20× (N.A. (Numerical Aperture) = 0.50), 40× (N.A. = 1.25), 60× (N.A. = 1.20), and 100× (N.A. = 1.40) objective lenses (Olympus). The fluorescence signals of GFP and YFP were mainly detected with a filter cube U-MYFPHQ (Olympus; excitation 490–500 nm; emission 515–560 nm), while that of CFP was detected with CFP-2432A (Semrock, Rochester, NY, USA; excitation 426–450 nm; emission 465–501 nm). Bright-field images were taken with differential interference contrast (DIC) optics. Unless otherwise specified, three independent plants were employed to count the amyloplast number per integument cell and measure the plastid plan area and stromule frequency and length.

### Western analysis

Total proteins were extracted from the whole pistils of Col, Ws, FtsZ1-GFP, FtsZ1-GFP-ox, and *minE* plants. Pistils were homogenized in a microcentrifuge tube containing a 5 mm Zirconia bead and ice-cold buffer (60 mm Tris-HCl [pH 8.0], 100 mm dithiothreitol, 2% (w/v) SDS, 15% (w/v) sucrose, 1 mm phenylmethanesulfonyl fluoride, 5 *µ*g L^−1^ leupeptin, 5 *µ*g L^−1^ chymostatin, and 5 *µ*g L^−1^ antipain) using a TissueLyser II (Qiagen, Hilden, Germany). Total protein was extracted from 1.0 mg (for FtsZ1) or 0.2 mg (for FtsZ1-GFP) fresh weight of pistils and subjected to SDS-PAGE (Nishihama et al. 2001). Then, FtsZ1 and FtsZ1-GFP proteins were detected by western blotting using anti-FtsZ1 antibody (provided by Prof. K. W. Osteryoung, Michigan State University) and Living Color GFP Monoclonal Antibody (Clontech, Mountain View, CA, USA), respectively (as primary antibodies), followed by horseradish peroxidase-linked anti-rabbit and anti-mouse antibodies (secondary antibodies; GE HealthCare, Tokyo, Japan).

### Generation of transgenic *ftsZ* line expressing stroma-targeted YFP

To monitor plastids in the integument cells of the *ftsZ* triple KO mutant (Schmitz et al. 2009), the plant transformation vector pSMAB-Z1TP-YFP (Chen et al. 2009) was employed. This vector carries a fusion of the N-terminal TP (90 aa) of FtsZ1 with the N-terminus of YFP (EYFP; Clontech Laboratories, Mountain View, CA), and a fusion of the N-terminal nuclear localization signal (NLS; 60 aa) of Cry2 with the N-terminus of CFP under the control of the CaMV *35S* promoter and the *NOS* terminator (*CaMV35Spro*::*TP-YFP*::*NOSter CaMV35Spro*::*NLS-CFP*::*NOSter*). The pSMAB-Z1TP-YFP vector was introduced into *A. tumefaciens*, which was then used to transform Arabidopsis plants by the floral dip method (Clough and Bent 1998). A total of 200 T_1_ lines were selected at the seedling stage on MS medium containing 4 *µ*g/L bialaphos (Meiji Seika, Tokyo, Japan). Of the 200 T_1_ lines, 10 lines showed high-level and stable YFP fluorescence, one of which (R39-3) was chosen for stereofluorescence microscopy (Leica FLIII, Heidelberg, Germany). The T_2_ and T_3_ progenies were used for subsequent characterization by fluorescence microscopy.

### Image analysis

The digital images were processed mainly using Adobe Photoshop CS3 or CS6 (Adobe Systems, San Jose, CA). ImageJ ver. 1.42q or 1.53a (National Institute of Health, Bethesda, MD) was used to measure plastid plan areas and stromule frequencies and lengths and to prepare movies. Stromule frequency represents the percentage of plastids emanating stromules (Kojo et al. 2009; Erickson et al. 2023). 150 plastids for Phase II and 100 plastids for Phase III were examined for each analysis. Stromule length represents the total length of stromules per plastid. Up to 50 stromule-emanating plastids were selected from the dataset of stromule frequency analysis and measured using the “segmented line” tool of ImageJ.

### Statistical analyses

Statistical analyses were performed with Microsoft Excel and GraphPad Prism 8 (GraphPad Software, San Diego, CA). In boxplots, the upper and lower boundaries represent the 3/4 and 1/4 lines, respectively, and the whiskers above and below the box represent 9/10 and 1/10 lines, respectively. The single horizontal line and “+” mark within the box represent the median and mean values, respectively, and the open circles outside the box represent outliers.

### Accession numbers

Sequence data from this article can be found in the GenBank/EMBL data libraries under accession numbers: At1g69390 (*AtMinE1*), At5g24020 (*AtMinD1*), At3g19720 (*ARC5*/*DRP5B*), At5g42480 (*ARC6*), At5g55280 (*AtFtsZ1-1*), At2g36250 (*AtFtsZ2-1*), and At3g52750 (*AtFtsZ2-2*).

## Supplementary Material

kiae314_Supplementary_Data

## Data Availability

The data underlying this article are available in the article and in its online supplementary material.

## Supplementary data

The following materials are available in the online version of this article.

**Supplementary Figure S1.** Amyloplast number in mature ovule integument cells of wild-type and five plastid division mutants of Arabidopsis.

**Supplementary Figure S2.** Measurement of the plastid plan area in mature ovule integument cells of wild-type (WT) and four plastid division mutants of Arabidopsis.

**Supplementary Figure S3.** Moderate variation in size and shape of amyloplasts in ovule integument cells of wild-type Arabidopsis plants and its comparison with that of chloroplasts in photosynthetic cells.

**Supplementary Figure S4.** Developmental dynamics of amyloplast morphologies in *minD*, *arc5*, and *arc6* mutants.

**Supplementary Figure S5.** Measurement of the stromule length in developing and mature ovule integument cells of wild-type (WT) and four plastid division mutants.

**Supplementary Figure S6.** Expression and localization of the FtsZ1-GFP fusion in transgenic Arabidopsis plants.

**Supplementary Figure S7.** Characterization of FtsZ ring formation in *FtsZ1-GFP* transgenic plants during amyloplast replication.

**Supplementary Figure S8.** FtsZ ring formation during amyloplast replication in wild-type and *minE*.V

**Supplementary Figure S9.** FtsZ ring formation during amyloplast differentiation in wild-type.

**Supplementary Figure S10.** DNA staining of integument cells of wild-type and *minE*.

**Supplementary Figure S11.** Characterization of amyloplast and stromule morphologies in the integument cells of the *ftsZ* mutant expressing stroma-targeted YFP.

**Supplementary Figure S12.** Stromule lengths and plastid numbers in developing and mature ovule integument cells of the *ftsZ* mutant.

**Supplementary Figure S13.** A working model of amyloplast replication events in wild-type and *minE* integument cells.

**Supplementary Video S1.** Time-lapse observation of a YFP-labeled amyloplast and its stromules in an integument cell of *minE*.

**Supplementary Video S2.** Time-lapse observation of YFP-labeled and highly induced stromules in giant amyloplasts of *ftsZ*.

**Supplementary Video S3.** Time-lapse observation of a YFP-labeled amyloplast and its stromules in an integument cell of *ftsZ*.

## Dive Curated Terms

The following phenotypic, genotypic, and functional terms are of significance to the work described in this paper:

arc5 Gramene: AT3G19720
arc5 Araport: AT3G19720

ARC6 Gramene: AT5G42480
ARC6 Araport: AT5G42480

minD Gramene: AT5G24020
minD Araport: AT5G24020

AtMinE1 Gramene: AT1G69390
AtMinE1 Araport: AT1G69390

integument AmiGo: PO:0020021

starch CHEBI: CHEBI:28017

ARC3 Gramene: AT1G75010
ARC3 Araport: AT1G75010

PARC6 Gramene: AT3G19180
PARC6 Araport: AT3G19180
