# Supplementary material for: Principles of amyloplast replication in the ovule integuments of Arabidopsis thaliana
Source: Plant Physiol. 2024 Jun 3;196(1):137–52. doi: 10.1093/plphys/kiae314 (PMC11376375; doi:10.1093/plphys/kiae314)
Supplement: kiae314_Supplementary_Data [file kiae314_supplementary_data.zip › Supplementary Material.pdf]

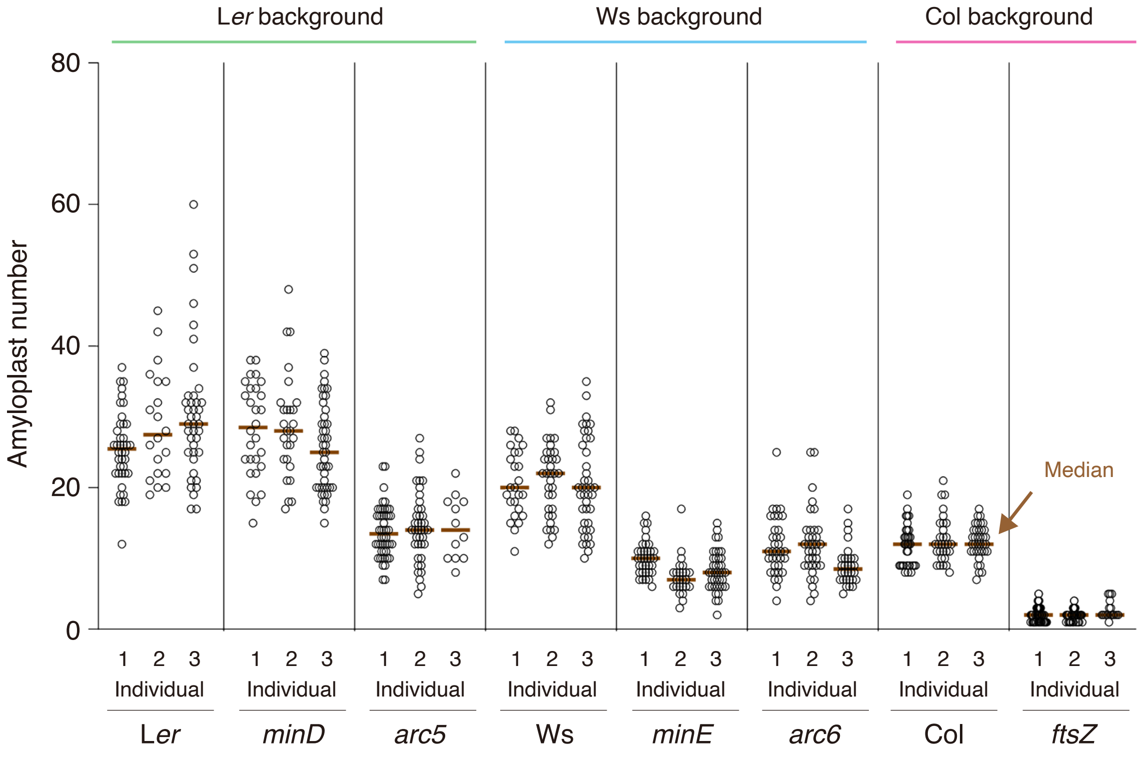

Supplementary Figure S1. Amyloplast number in mature ovule integument cells of wild-type and five plastid division mutants of Arabidopsis. The plots of the data of Fig. 1L are presented at the individual plant level.

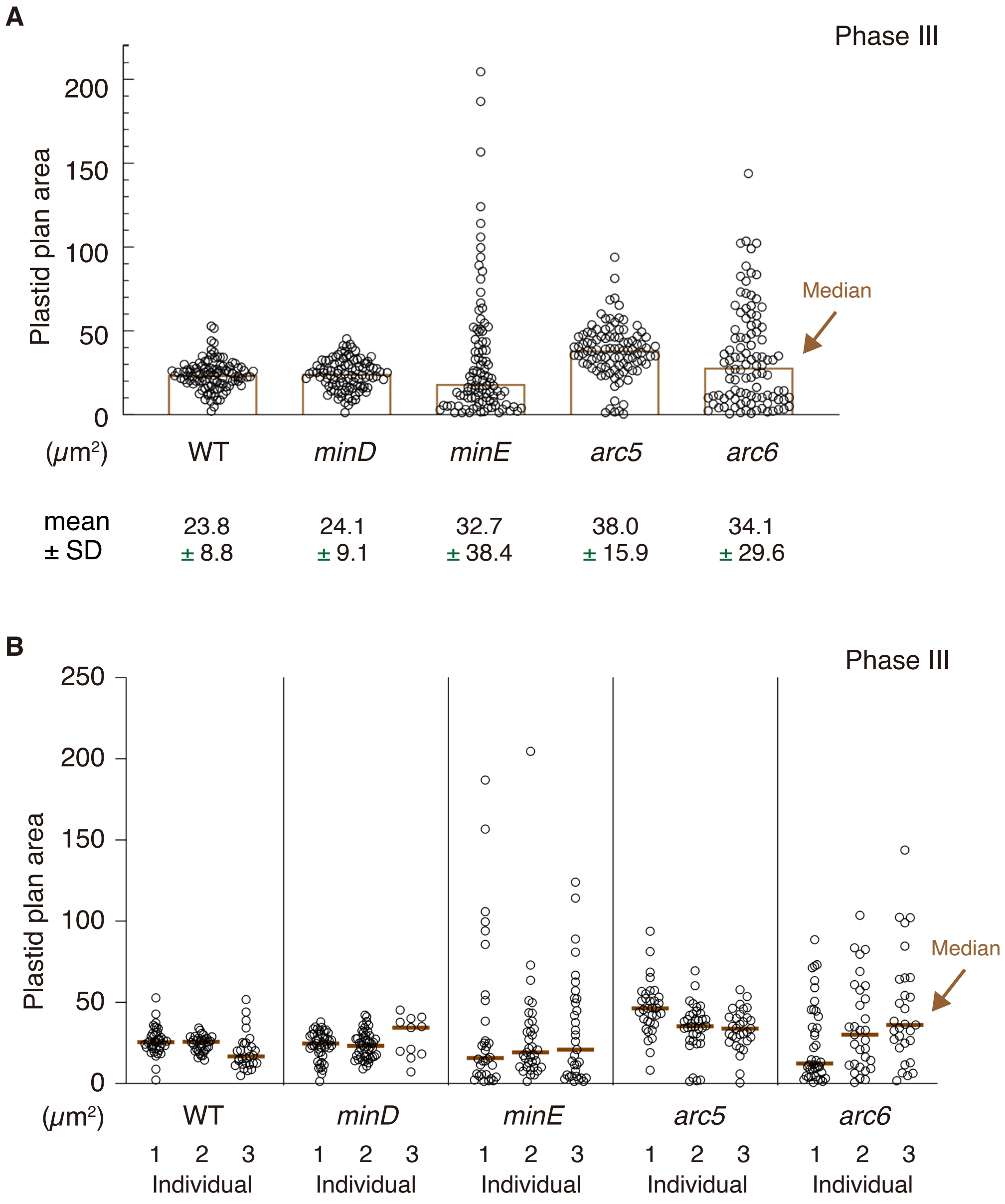

Supplementary Figure S2. Measurement of the plastid plan area in mature ovule integument cells of wild-type (WT) and four plastid division mutants of Arabidopsis. Integument cells of Arabidopsis plants expressing stroma-targeted fluorescent proteins were observed by fluorescence microscopy, and the images were analyzed by ImageJ. (A) The plots of the data of Fig. 2B (n = 100 plastids, from three independent plants) are shown with median and mean ± SD values (μm^2). (B) The plots of the data (A) at the individual plant level.

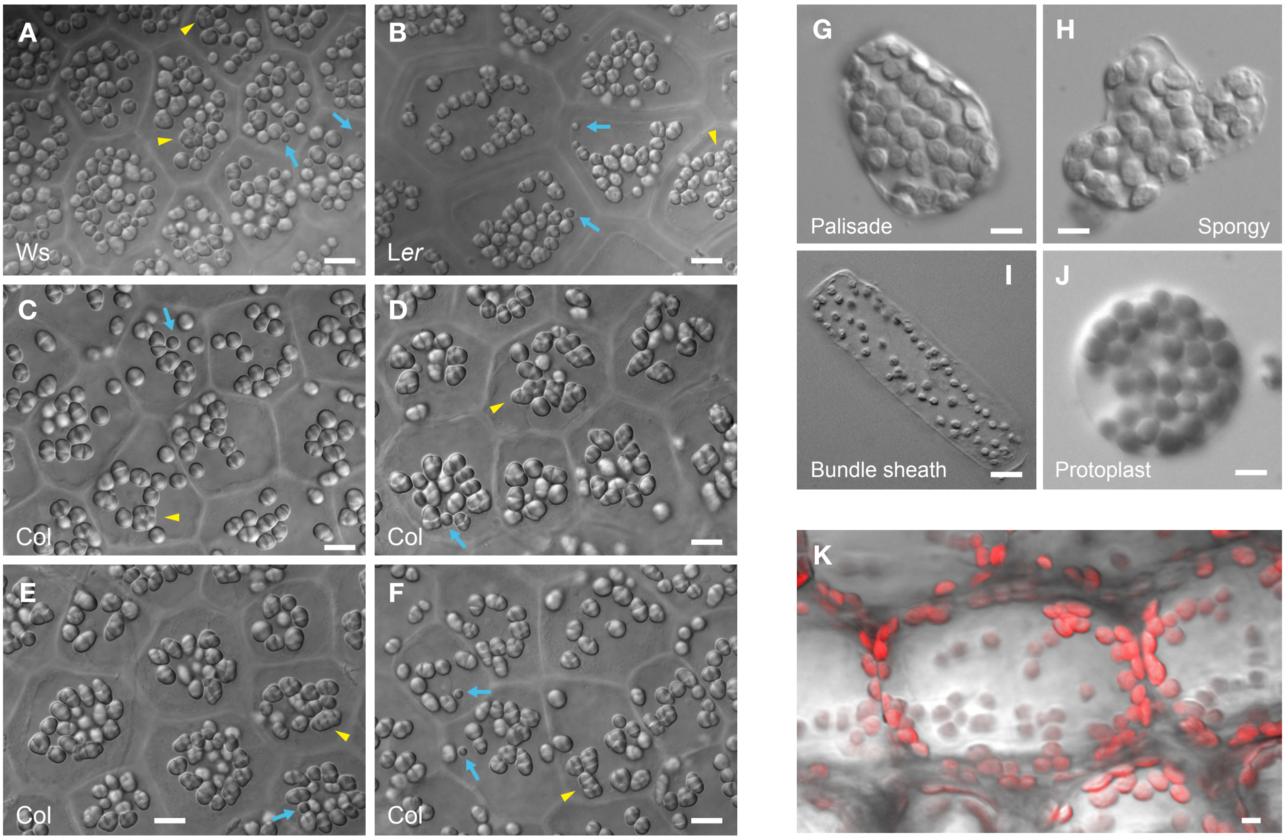

Supplementary Figure S3. Moderate variation in size and shape of amyloplasts in ovule integument cells of wild-type Arabidopsis plants and its comparison with that of chloroplasts in photosynthetic cells. (A–F) Amyloplasts in ovule integument cells of Ws (A), L*er* (B), and Col (C–F) plants. Arrows and arrowheads indicate small and large amyloplasts, respectively. (G–J) Chloroplasts in palisade (G), spongy (H), bundles sheath (I), and protoplast (J) cells from mature leaves of Col plants. (K) Chloroplasts in hypocotyl cortex cells of Col plants. Chlorophyll autofluorescence signals are colored in red. Scale bars: 10 µm (all).

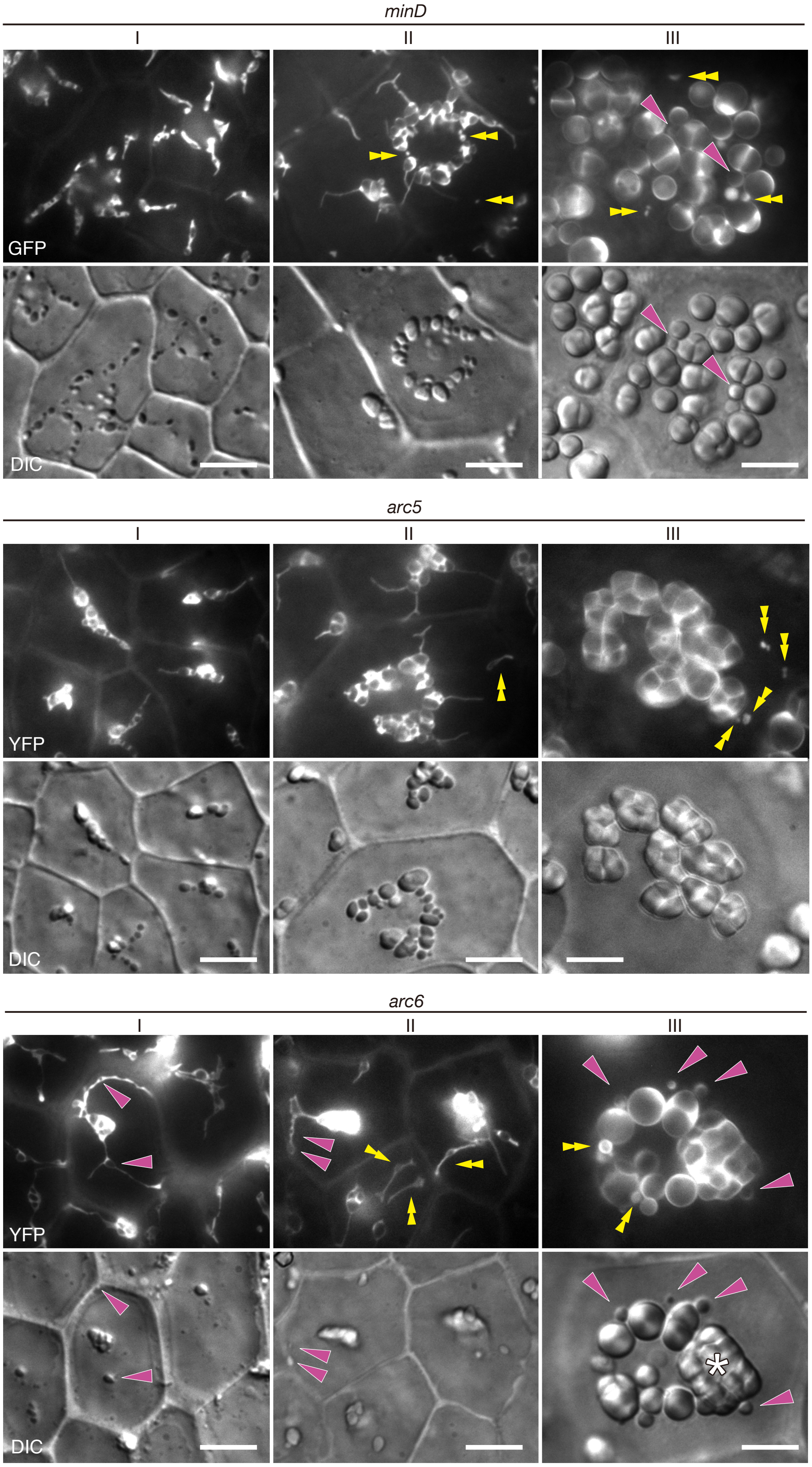

Supplementary Figure S4. Developmental dynamics of amyloplast morphologies in *minD*, *arc5*, and *arc6* mutants. Integument cells of *minD*, *arc5*, and *arc6* mutants expressing stroma-targeted fluorescent proteins were characterized by fluorescence microscopy. Fluorescence and differential interference contrast (DIC) images are shown. Asterisks indicate giant amyloplasts, and single and double arrowheads indicate the position of tiny starch grains and starchless plastids, respectively. Scale bars: 10 μm (all).

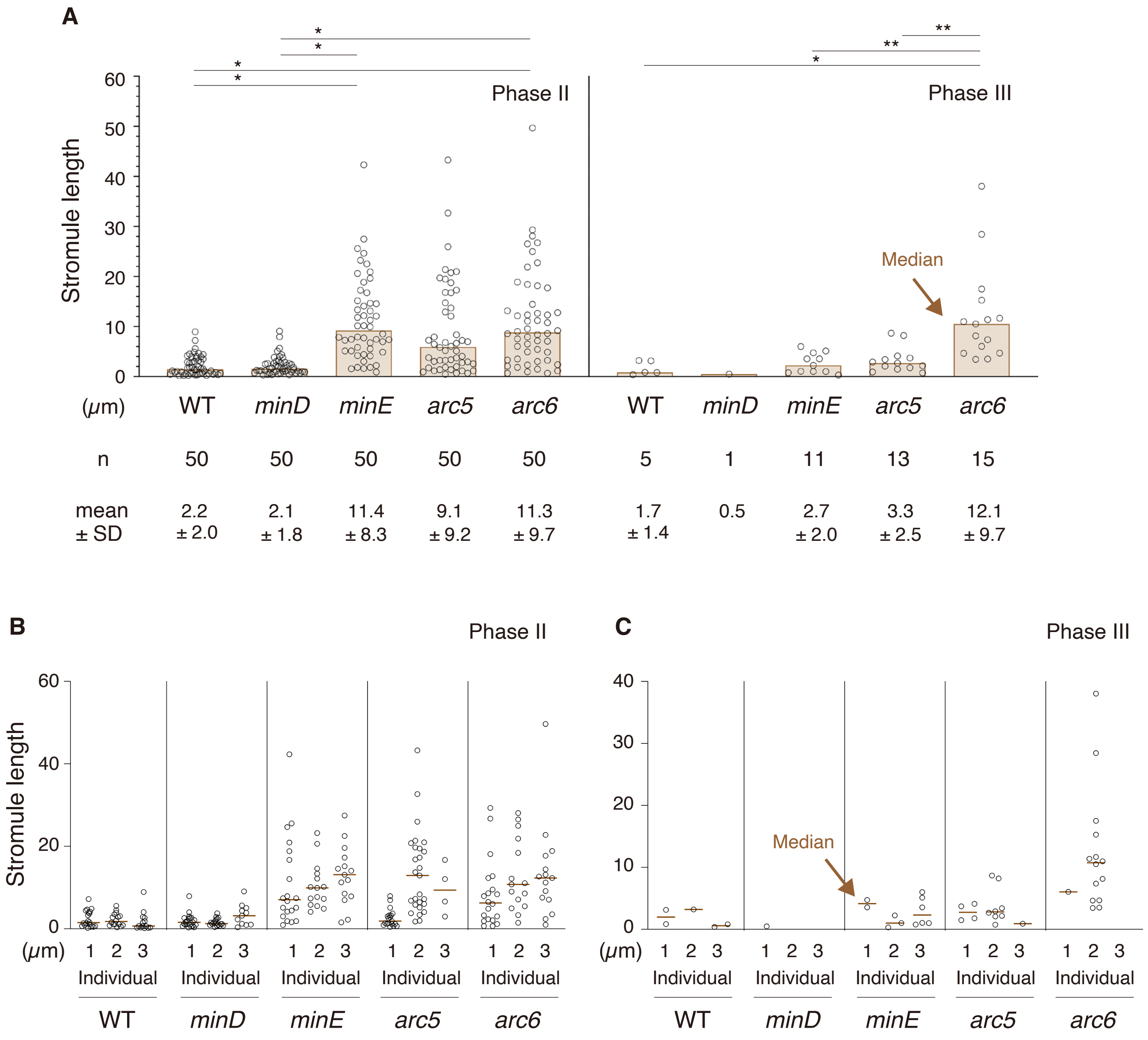

Supplementary Figure S5. Measurement of the stromule length in developing and mature ovule integument cells of wild-type (WT) and four plastid division mutants. Integument cells of Arabidopsis plants expressing stroma-targeted fluorescent proteins were observed by fluorescence microscopy, and the images were analyzed by ImageJ. (A) The plots of the data (n = up to 50 plastids; derived from three independent plants) are shown with median and mean ± SD values (μm). The differences between plants were analyzed by a nested one-way ANOVA with Tukey's multiple comparison test (* $p < 0.05$; ** $p < 0.01$; only samples with significant differences are indicated). (B, C) The plots of the data (A) at the individual plant level for Phase II (B) and Phase III (C). Note that, in the analysis of *minD*, only 1 of 100 Phase III amyloplasts from three plants had stromules. Two plants showed no stromules in the analyzed amyloplasts.

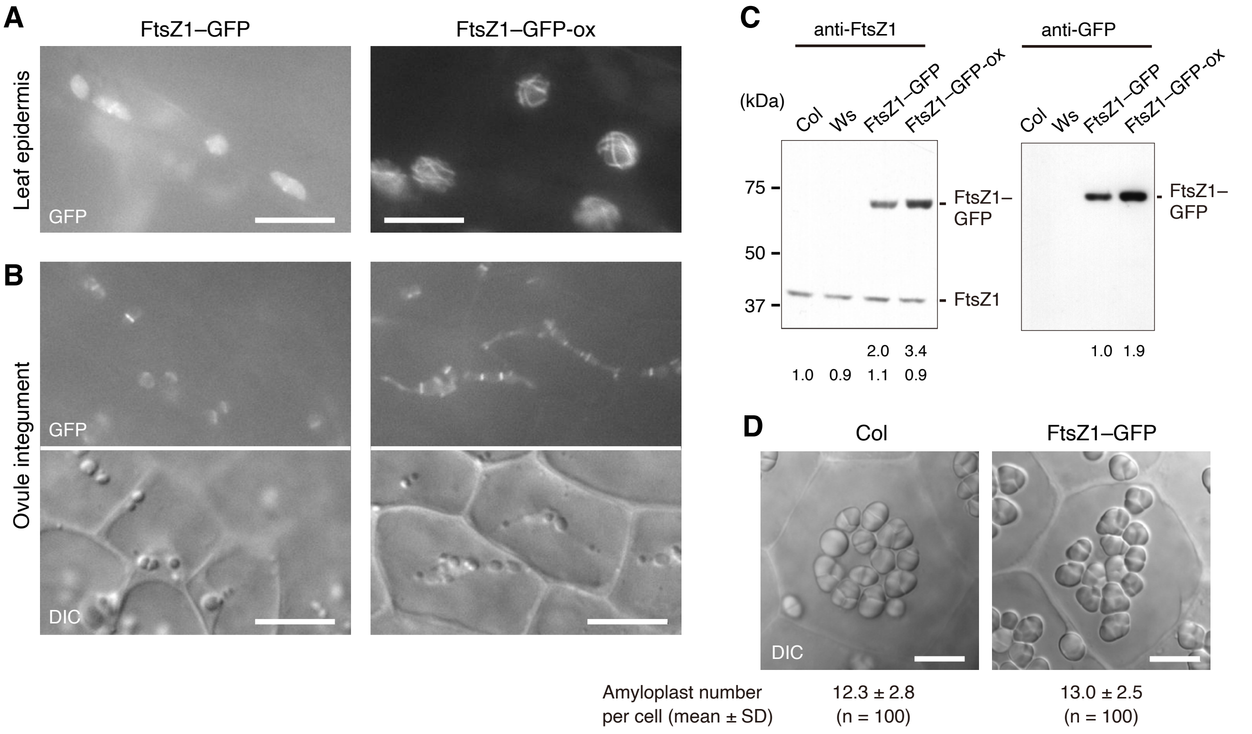

Supplementary Figure S6. Expression and localization of the FtsZ1-GFP fusion in transgenic Arabidopsis plants. (A, B) Detection of GFP signals in the leaf epidermis (A) and ovule integument (B) of transgenic Arabidopsis plants (Col background) expressing *FtsZ1-GFP* moderately (FtsZ1-GFP line) or strongly (FtsZ1-GFP-ox line) (Fujiwara et al., 2009). The same individual plants were used for (A) and (B). (C) Western blot analysis of FtsZ1-GFP in pistils using anti-FtsZ1 (left) and anti-GFP (right) antibodies. Col and Ws were used as negative controls. Relative band intensity in the images is also shown. (D) Phenotypes of mature (Phase III) amyloplasts in the integument cells of wild-type (Col) and *FtsZ1-GFP* transgenic plants. Differential interference contrast (DIC) images and amyloplast number per integument cell are shown. Scale bars: 10 µm (A, B, and D).

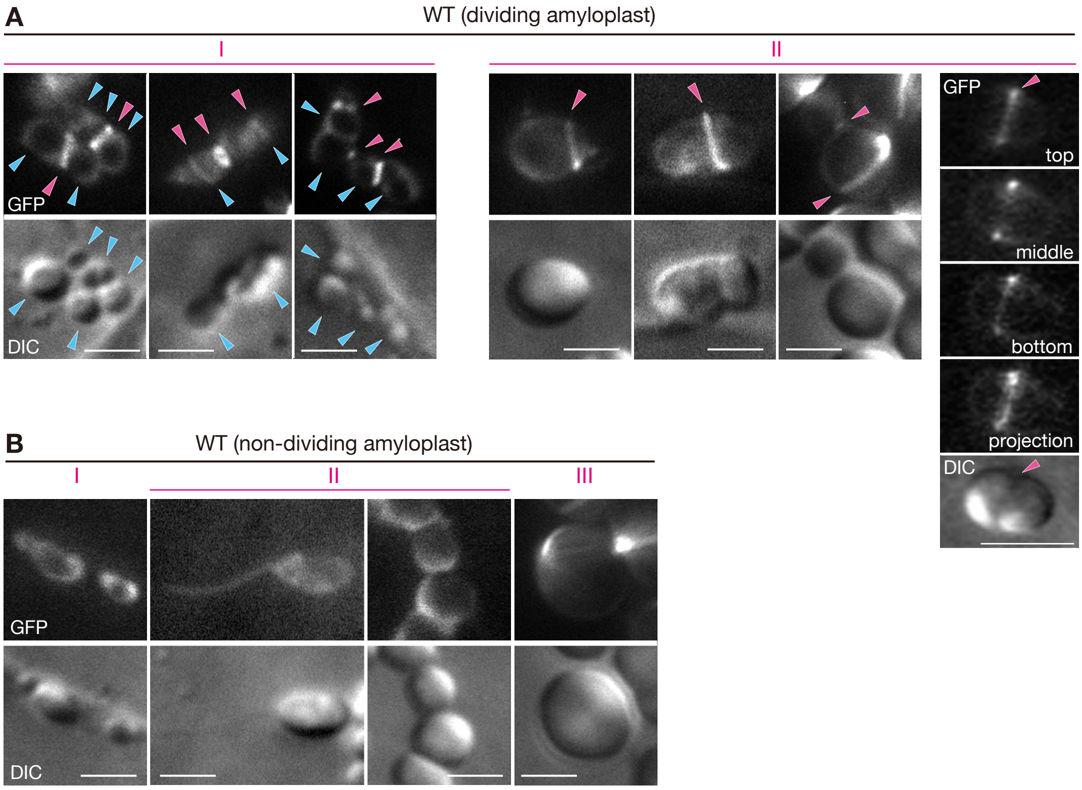

Supplementary Figure S7. Characterization of FtsZ ring formation in *FtsZ1-GFP* transgenic plants during amyloplast replication. (A) Formation of single to multiple FtsZ rings at the constriction and non-constriction sites of amyloplasts. (B) Localization patterns of FtsZ1-GFP in amyloplasts in the non-proliferating state. Cyan and magenta arrowheads indicate the positions of starch grains and FtsZ rings, respectively. Scale bars: 3 µm (all).

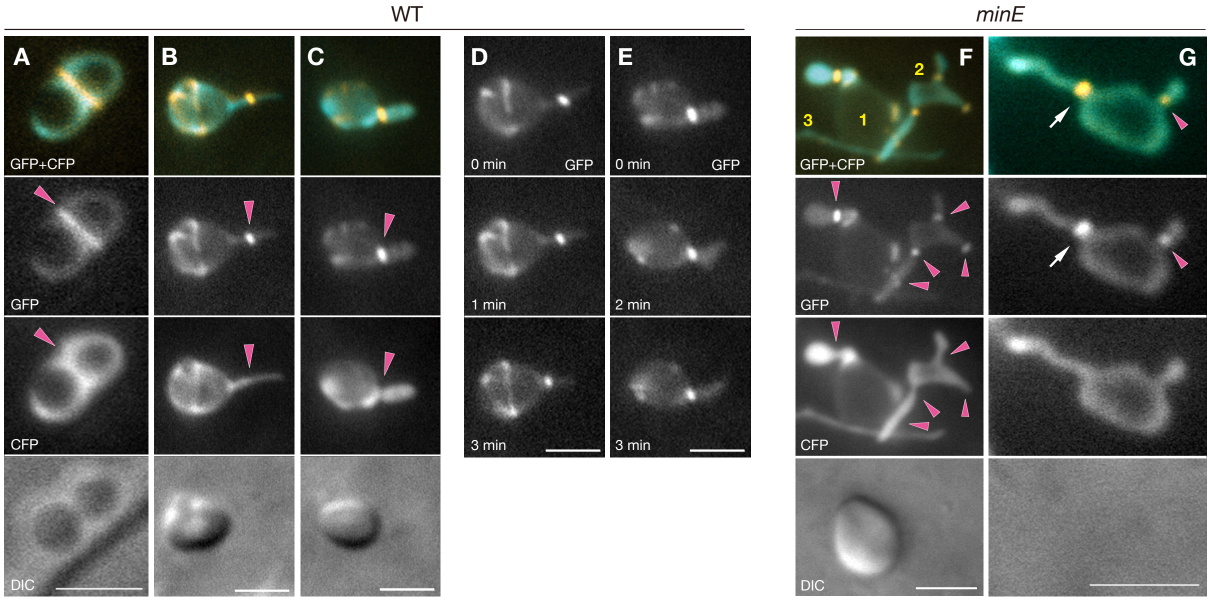

Supplementary Figure S8. FtsZ ring formation during amyloplast replication in wild-type and *minE*. Integument cells of wild-type (WT) and *minE* plants expressing FtsZ1-GFP and stroma-targeted CFP were microscopically characterized. (A–E) WT. (F, G) *minE*. In (B) and (C), amyloplasts with a single FtsZ ring at constriction or non-constriction sites of stromules are shown. In (D) and (E), single FtsZ rings localized in the stromules were observed by time-lapse fluorescence microscopy. The time points captured in the images are indicated. Note that GFP images in (B) and (C) are reused in (D) and (E), respectively. Arrowheads indicate the positions of FtsZ rings, while arrows indicate a non-constriction site of a stromule associated with an FtsZ ring. In (F), number indicates the presence of three independent plastids in the image. In all the merged images, signals of CFP (cyan) and GFP (orange) are pseudo-colored. Scale bars: 3 µm (all).

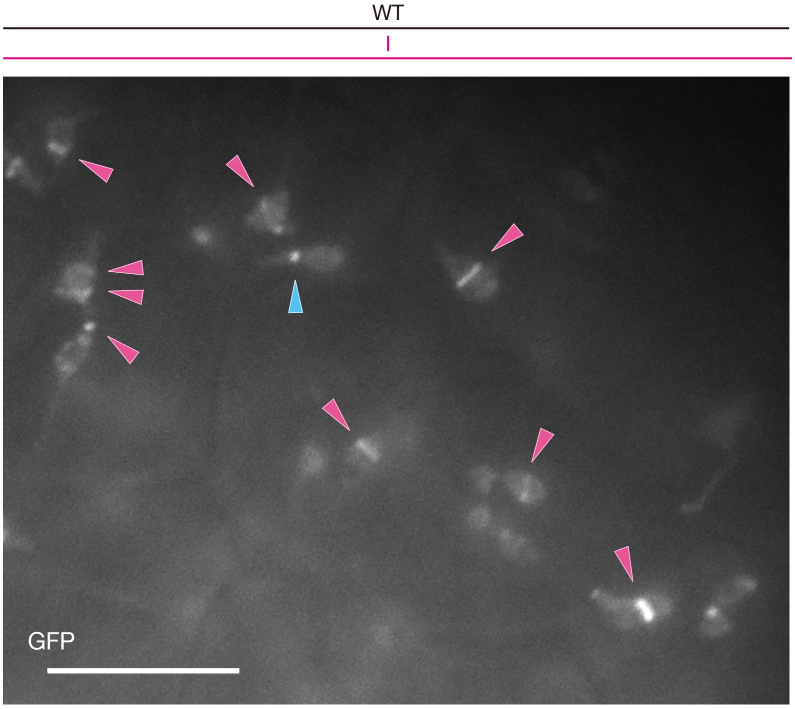

Supplementary Figure S9. FtsZ ring formation during amyloplast differentiation in wild-type. Integument cells of wild-type (WT) plants expressing both FtsZ1-GFP and stroma-targeted CFP were characterized using microscopy. An overview image of differentiating amyloplasts (Phase I) in a cell is presented. Magenta and cyan arrowheads indicate the FtsZ ring located at the plastid body and the stromule, respectively. Scale bar: 10 µm.

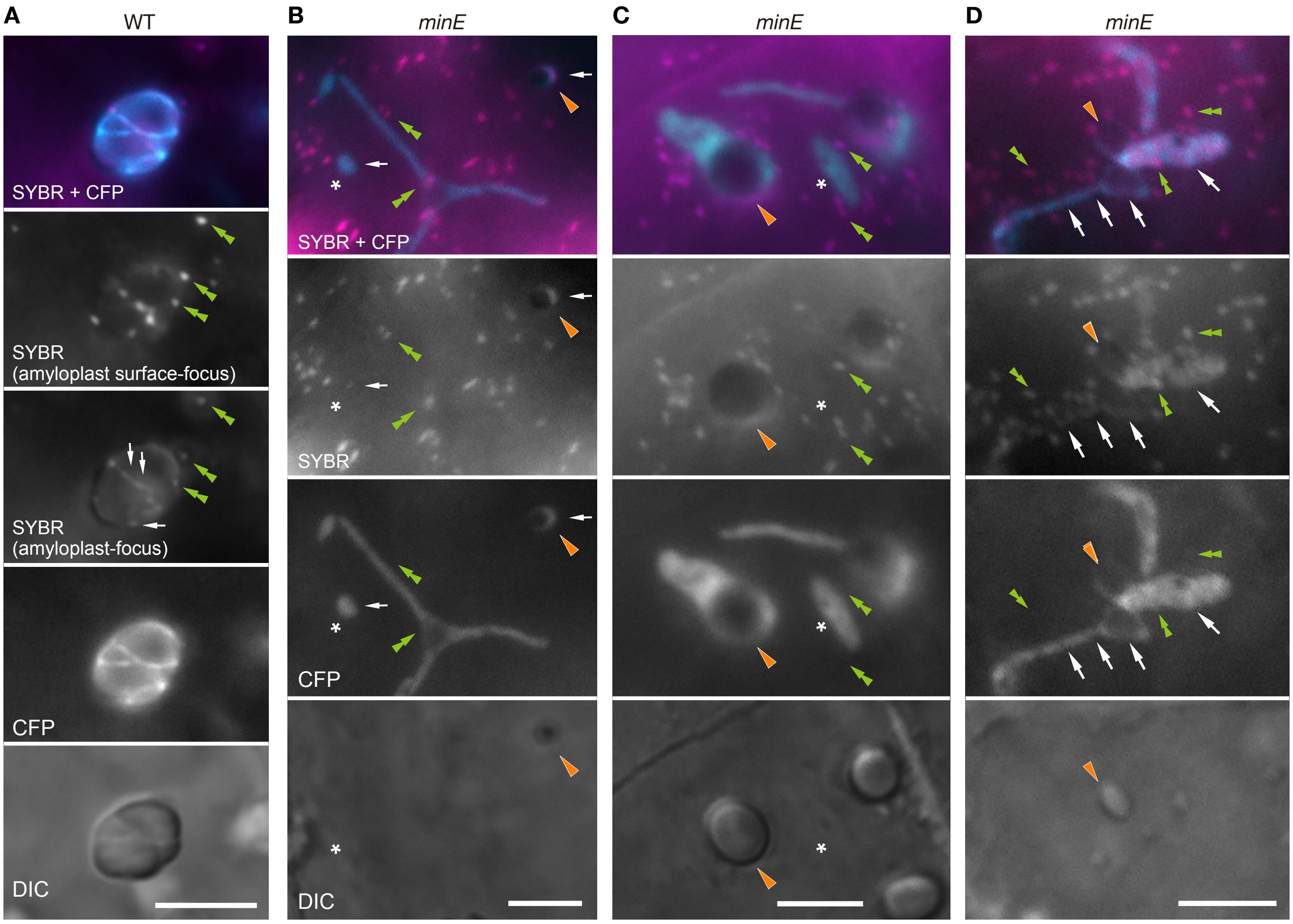

Supplementary Figure S10. DNA staining of integument cells of wild-type and *minE*. Developing ovules of wild-type (WT) and *minE* plants expressing stroma-targeted CFP was expressed were chemically fixed, stained with SYBR Green, and observed by fluorescence microscopy. (A) WT. A WT-segregant from *minE* × TP-CFP was investigated to distinguish between mitochondrial and plastid nucleoids. (B–D) *minE*. (B) Detection of nucleoids in small amyloplasts or plastids. (C) The absence of SYBR Green signals in a putative starchless plastid. (D) Detection of nucleoids in the plastid body and the stromule of a developing amyloplast. SYBR Green (magenta) and CFP (cyan), SYBR Green, CFP, and DIC images are shown. Single arrowheads and asterisks indicate the position of starch grains and putative starchless plastids, respectively. Arrows and double arrowheads indicate fluorescence signals from putative plastid and mitochondrial nucleoids, respectively. Scale bars: 5 µm (all).

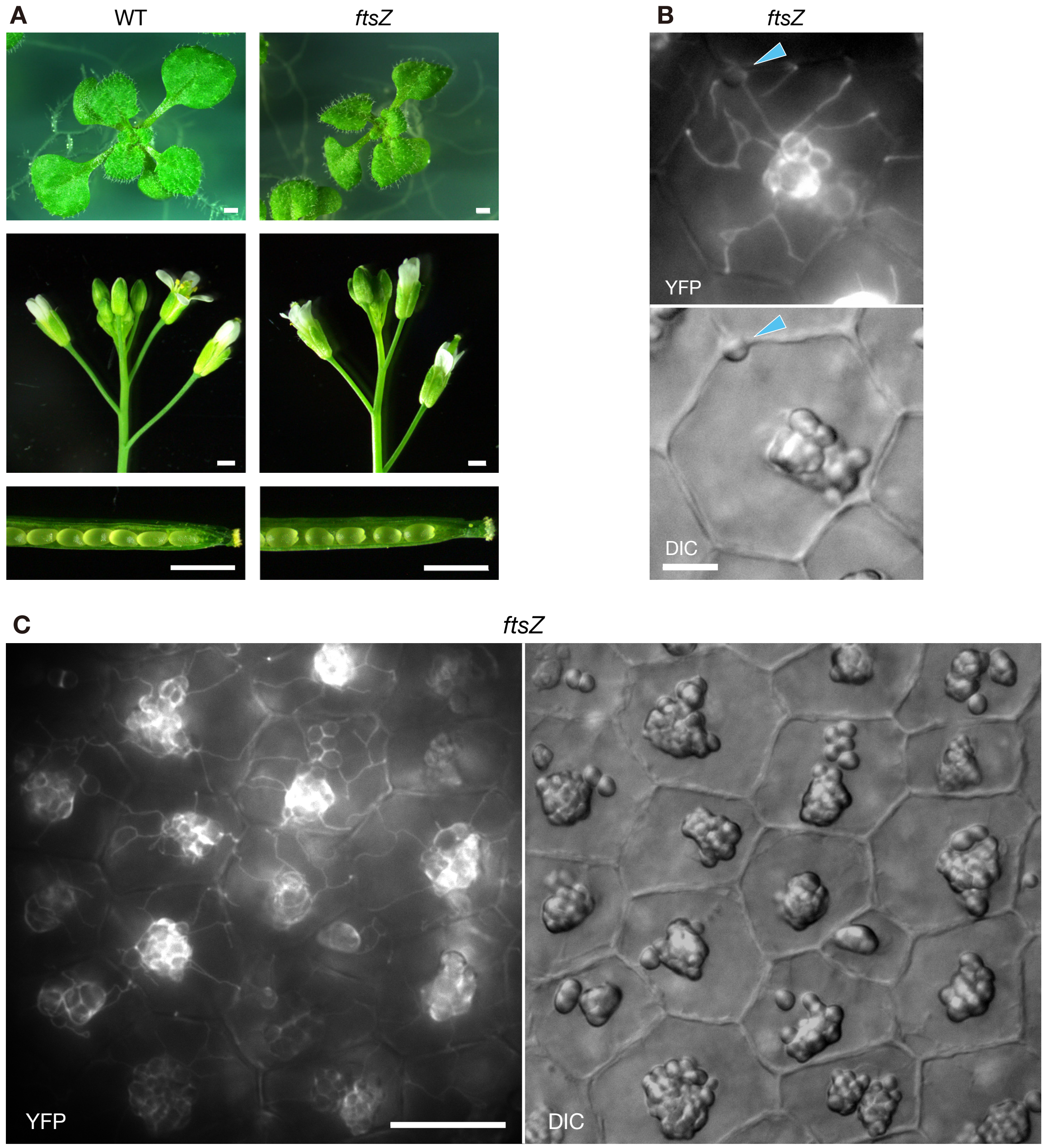

Supplementary Figure S11. Characterization of amyloplast and stromule morphologies in the integument cells of the *ftsZ* mutant expressing stroma-targeted YFP. (A) Growth and reproduction of the *ftsZ* mutant. The phenotype of seedlings (top), inflorescences (middle), and seeds (bottom) of wild-type (WT) and *ftsZ* are shown. (B) Giant amyloplast fromation with hyper-activation of stromules. Arrowheads indicate starch grains within a giant amyloplast-derived stromule. (C) Presence of stromules until the later stages of amyloplast development in integment cells. Scale bars: 1 mm (A), 10 µm (B), and 30 µm (C).

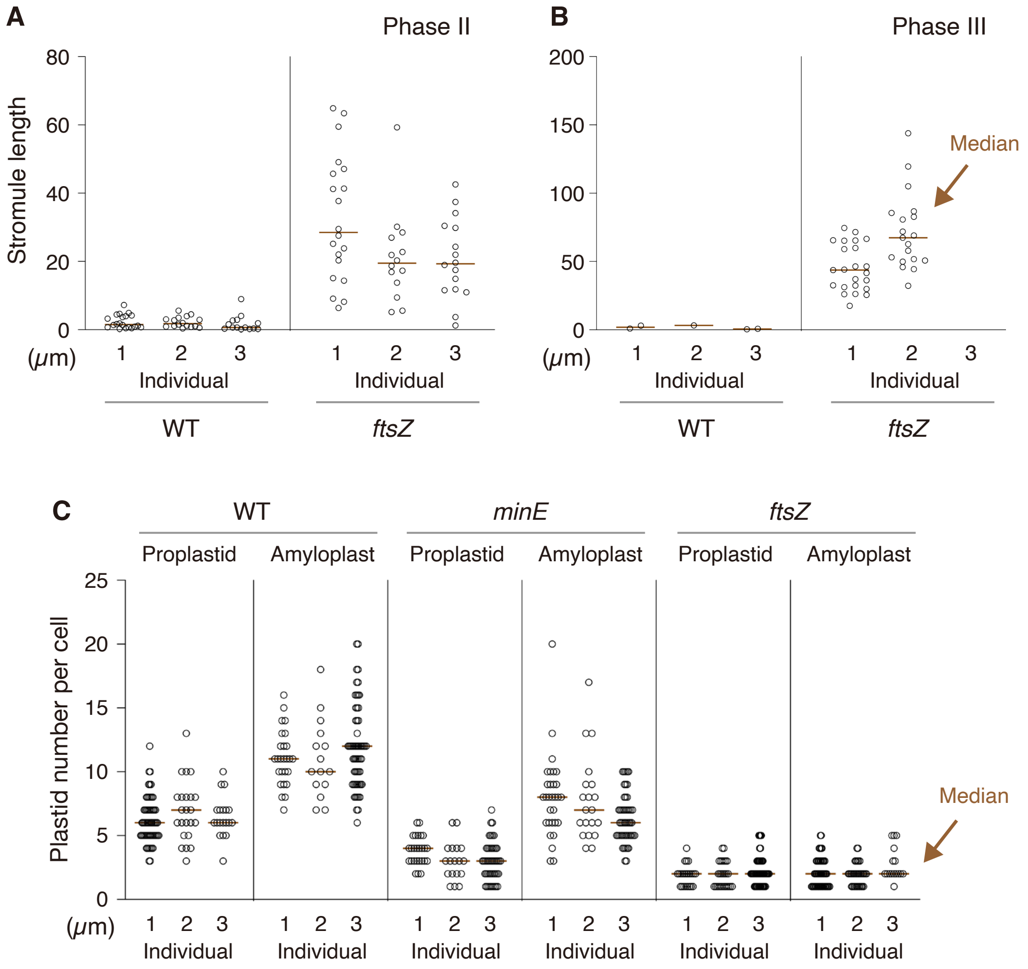

Supplementary Figure S12. The stromule length and plastid number in developing and mature ovule integument cells of the *ftsZ* mutant. Integument cells of Arabidopsis plants expressing stroma-targeted fluorescent proteins were analyzed. (A, B) Measurement of stromule length. The plots of the data of Fig. 5B for Phase II (A) and Phase III (B) at the individual plant level are shown. (C) The number of proplastids and amyloplasts per cell. The data of Fig. 5C at the individual plant level are shown. Note that the sum of analyzed cells for proplastid counting from three *minE* plants (individuals 1, 2, and 3) is 91, while the others are 100, in this panel.

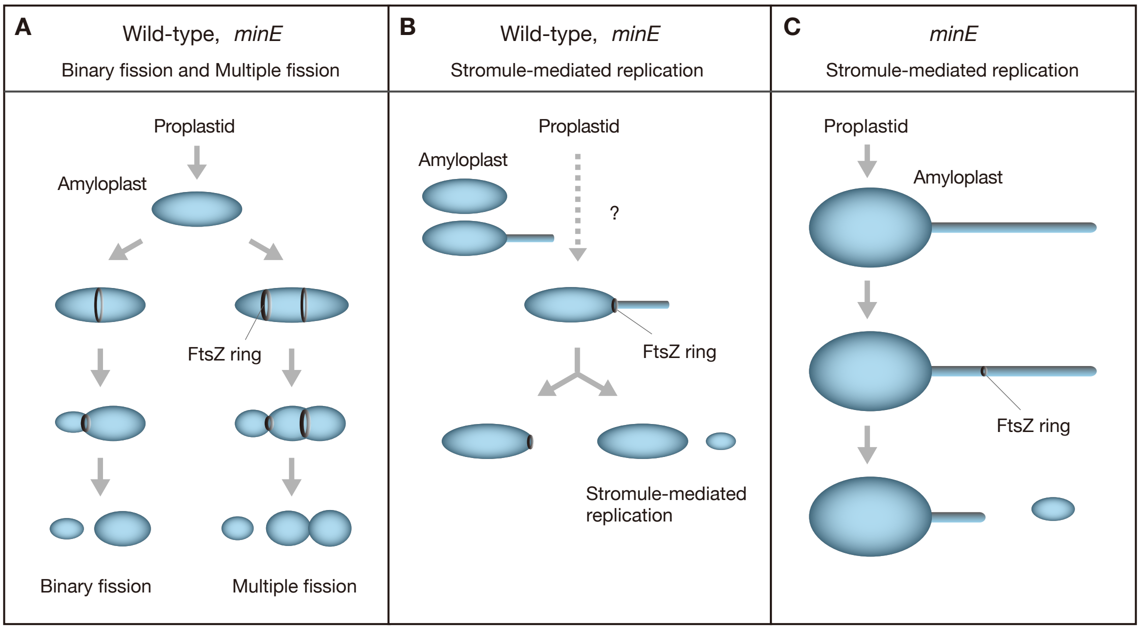

Supplementary Figure S13. A working model of amyloplast replication events in wild-type and *minE* integument cells. (A) Binary fission and multiple fission of amyloplasts in wild-type and *minE*. (B) Stromule-mediated replication by fission at the stromule neck in wild-type and *minE*. Note that processes that occur upstream of FtsZ ring formation at stromules are not fully resolved. (C) Stromule-mediated replication by fission at random site(s) of stromules found exclusively in *minE*. Schematic models with information on the FtsZ ring in plastid bodies or stromules are presented. In these models, information on the number of amyloplast (plastid) fission sites, spatial distribution of the FtsZ ring, and variation in the size and shape of amyloplasts and stromules has been reduced for simplicity.
