# Supplementary material for: Principles of amyloplast replication in the ovule integuments of Arabidopsis thaliana
Source: Plant Physiol. 2024 Jun 3;196(1):137–52. doi: 10.1093/plphys/kiae314 (PMC11376375; doi:10.1093/plphys/kiae314)
Supplement: kiae314_Supplementary_Data [file kiae314_supplementary_data.zip › Supplementary Video Legends.pdf]

Supplementary Video Legends

Supplementary Video S1. Time-lapse observation of a YFP-labeled amyloplast and its stromules in an integument cell of *minE*. Fluorescence signals of stroma-targeted YFP were traced by time-lapse fluorescence microscopy over a period of 45 min. 45 min corresponds to 4.5 s in this movie.

Supplementary Video S2. Time-lapse observation of YFP-labeled and highly induced stromules in giant amyloplasts of *ftsZ*. Fluorescence signals of stroma-targeted YFP were traced by time-lapse fluorescence microscopy over a period of 13 min. 13 min corresponds to 3.7 s in this movie.

Supplementary Video S3. Time-lapse observation of a YFP-labeled amyloplast and its stromules in an integument cell of *ftsZ*. luorescence signals of stroma-targeted YFP were traced by time-lapse fluorescence microscopy over a period of 40 min. 40 min corresponds to 8.0 s in this movie.
