# Supplementary material for: Principles of amyloplast replication in the ovule integuments of Arabidopsis thaliana
Source: Plant Physiol. 2024 Jun 3;196(1):137–52. doi: 10.1093/plphys/kiae314 (PMC11376375; doi:10.1093/plphys/kiae314)
Supplement: kiae314_Supplementary_Data [file pp.01597.2023-s07.pdf › pp.01597.2023-s07.pdf]

Plant Physiology®

Principles of amyloplast replication in the ovule integuments of Arabidopsis thaliana

Makoto Fujiwara, Yasushi Yoshioka, Yusuke Kazama, Tomonari Hirano, Yasuo Niwa, Takashi Moriyama, Naoki Sato, Tomoko Abe, Shigeo Yoshida, and Ryuuichi Itoh

Review Timeline:

Submission Date:	24-Oct-2023
Editorial Decision:	24-Nov-2023
Revision Received:	01-Mar-2024
Editorial Decision:	13-Mar-2024
Revision Received:	09-Apr-2024
Editorial Decision:	23-Apr-2024
Revision Received:	30-Apr-2024
Accepted:	01-May-2024

24-Nov-2023

Dr. Makoto T Fujiwara
Jochi Daigaku
Department of Materials and Life Sciences
Tokyo 102-8554
Japan

RE: Principles of amyloplast replication in the ovule integuments of Arabidopsis thaliana

Dear Dr. Fujiwara:

We have received two reviews of your manuscript, which you may view at the end of this letter. On the basis of the advice received and my own evaluation, I believe that this article may ultimately be acceptable for publication in Plant Physiology but that additional revision, data
analysis and experimental work is first required.

When you are ready to submit your revision, click the below link and find the red arrow under your Author Tasks heading for this revision submission.

If you want to visit this version of the manuscript, you may click the link below and then click on the folder that reads "Post Decision Manuscripts".

Link Not Available

When uploading the revised version of this article, please be sure to include as complete an answer as possible to the referees' criticisms during the first round of reviews in the "response to reviewers" section.

Plant Physiology considers a revision for up to 60 days after a manuscript has been provisionally accepted; limited extension of this timeframe is at the discretion of the Monitoring Editor. A reminder notice will be sent to you 2 weeks before the deadline, at which time you may choose to contact the editorial office to request an extension; if so, please provide a justification for your request. After the expiration of this deadline, the article would need to be submitted as a resubmission and would be handled as such.

Thank you very much for giving us an opportunity to review this work. I look forward to receiving the next version.

Sincerely,

Editor, Plant Physiology

Want to add this revision deadline to your calendar? Click below!

-- Reviewer comments:
Reviewer #1 (Comments for the Author):

Chloroplasts originated from a cyanobacterial endosymbiont and proliferate by binary fission in plant cells. Previous studies showed that chloroplast division is performed by constriction of a ring-like division machinery at the division site which is composed of several proteins including FtsZ of cyanobacterial origin and the dynamin-related protein ARC5. In addition, several proteins such as ARC6, MinD, and MinE have been shown to regulate FtsZ ring formation and positioning, which is the first step of the assembly of the division machinery.

Vascular plants have developed a complex system of cellular differentiation, in which plastids also differentiate into several types of non-photosynthetic plastids, in addition to photosynthetic chloroplasts. The current understanding of the mechanism of plastid division is mostly based on characterization of leaf chloroplasts. In contrast, some studies have shown that a certain types of plastids undergo multiple fission. In addition, in some mutants of the plastid division genes, accelerated formation of stromules

and proliferation of plastids were observed, raising a possibility that nonphotosynthetic plastids likely proliferate through formation of stromules in addition to division of the main plastid body. However, the nature and mechanisms of such unconventional forms of plastid division has remained unknown.

In this study, the authors have examined the course of proliferation of plastids in Arabidopsis thaliana ovule integuments where proplastids differentiate into amyloplast in the wild tyoe and some mutants of plastid division genes. Based on the seriese of observations, the authors conclude that amyloplast during development proliferate by three ways, binary fission, multiple fission, and stromule-mediated fission, all of which are mediated by FtsZ ring formation.

I think the observations are well presented and mostly interpreted soundly except. However, I still have two major concerns as below, and the MS lacks the necessary information to address these.

Major points:
1. I agree that stromules can undergo fission in a manner dependent on the FtsZ ring, as demonstrated by the results. However, I am curious about the frequency of this event in the wild type.

Regarding the rare presence of very small poorly developed plastids (Fig. S5) and the FtsZ ring at the neck of stromules in the wild type (Fig. 3B; Fig. S4A-E), what is the frequency at which these are observed? The statements of "minor FtsZ1 ring formation at stromules" (L253) and "the presence of stromules with a deep constriction and that of small plastids or amyloplasts (1-2 μm width) in mature integument cells (L258) lack quantifiability. The exact quantification is likely difficult technically but the author should provide rough estimation of the frequency of FtsZ ring associated with stromules and the small plastids in the wild type (e.g. the rato of the small plastids and developed amyloplasts or FtsZ ring formed in the plastid body and that associated with stromules or the maximum estimated values of them, etc.).

If these are very rare in the wild type compared to the minE mutant, I think that the conclusion of the MS (L39-41; L260-263; Fig. S7B) should be changed. That is, in the wild type, formation of FtsZ ring associated with stromules (and fission of plastid there) is not complete but considerably suppressed rather than "wild-type amyloplasts adopt three modes of replication, binary fission, multiple fission, and stromule-mediated fission, via multi-way placement of the FtsZ1 ring."

2. Do stromules and the newly generated tiny plastids via stromule fission contain nucleoids? I recommend that the authors examine whether stromule fission is capable of generating plastids with genomes by staining the samples with a fluorescent dye. This does not necessarily require live imaging, but simply the examination of the presence of DNA in stromules and the tiny plastids likely produced through stromule fission.

Minor points:
3. This is not mandatory but the results in Table1 is better to presented as a graph.

4. The statement in L157-159, "Wild-type cells contained round to ellipsoidal amyloplasts of relatively homogeneous size, although this homogeneity was less stringent than that of leaf chloroplasts (Fig. 1F, G)" lacks supporting data or references regarding the leaf chloroplasts.

5. I cannot understand the argument (L279-281) "We presumed that while the stromule can undergo narrowing without FtsZ1, the formation of FtsZ1 rings in minE stromules facilitate the constriction process." I think that this statement is not deduced from the results.

6. Similarly, the statement in L283-285, 'Nevertheless, minE mutant cells showed a shift towards stromule-mediated replication, and this mode of replication showed serious impairment in terms of FtsZ ring placement and FtsZ-dependency,' appears to conflict with the later result regarding 'FtsZ-dependency.' In fact, based on the subsequent results (Fig. 5), the authors conclude that FtsZ is indispensable for stromule fission (L347-349).

7. The argument in L302-304, "Thus, stromule stability underlies the formation, placement, and/or function of the FtsZ1 ring at stromules, although FtsZ ring relocation in plastids was observed for the first time in this study (Supplemental Fig. S4)" lacks a basis and is merely speculative.

8. In L469, TGD should be spelled out. Is it referring to "trigalactosyl-diacylglycerol"?

9. In L519, is "chloroplast" plastid or amyloplast?

10. It should be referred to as FtsZ ring instead of "FtsZ1-ring" (L41 and others). I understand that the authors observed FtsZ ring by expressing FtsZ1-GFP but "FtsZ1-ring" might confuse the readers by implying the existence of a specialized FtsZ ring that does not contain FtsZ2.

Reviewer #2 (Comments for the Author):

Fujiwara et al. have established the Arabidopsis ovule integument as a new system to study amyloplast replication, providing an interesting addition to crop studies. This is a useful system because it enables comparisons of chloroplast and amyloplast mechanisms in the same Arabidopsis plant, taking advantage of all the molecular and transgenic resources already available. The Arabidopsis ovule integument has compound and simple starch granules in the amyloplasts, contrasting with the solely simple starch granules in chloroplasts. This manuscript describes how the amyloplast and chloroplast division in Arabidopsis differ in regulation, by characterising the amyloplast phenotypes of plastid division mutants, focusing mainly on minE and ftsZ1, mutants in two major components of the plastid division machinery.

The authors demonstrated that wild-type amyloplasts have three mechanisms of division: binary fission, multiple fission and surprisingly, stromule-mediated fission. The stromules have a dynamic behaviour. The authors found that the minE phenotype differs in amyloplasts compared to what is known in chloroplasts, in that amyloplast division is not completely impaired. The minE amyloplasts can still divide but the resulting amyloplasts are heterogenous in size and starch content. minE mutants have an increase in stromules but the stromules are less dynamic than in wild type, and they have abnormal division sites, demonstrated by FtsZ1 localisation. FtsZ1 is essential for amyloplast division, with ftsz1 mutants having larger and fewer amyloplasts than wild type, with more and longer stromules.

This is an interesting manuscript that sheds new light on the roles of known players of plastid division, and especially the role of stromules which was previously unknown. The findings will be interesting for those working on plastids and starch in both Arabidopsis and in crops. However, to make this study more robust and presentable, more information is needed on the experimental set-up, with clarification on how many biological replicates were used. Throughout, the authors indicate how many cells were used but it is unclear from how many different plants these originate, and this would also impact the statistics and data presentation. The authors also make quantitative statements of comparison, but do not always show specific results that these statements are based on. The main conclusions based on the microscopy are interesting, but would be made more convincing with proper quantitative results. Some of the conclusions would also benefit from some more clarification. I highlight some of these specific points in the major points below.

Major points
1. Instances where there are quantitative statements made but the data is not shown:
a. Line 169: '50% fewer amyloplasts'
b. Line 268 - 269: The authors mention that Ftsz1 rings were found in stromules in minE at higher frequency than in the wild type. Therefore it would be helpful if there was a result that showed this, through quantification or an overview image.
2. Instances where biological replicates should be explained more clearly:
a. Table 1 - are all these cells from 1 plant or several plants?
b. Figure 2, both in the results and the materials and methods.
i. In figure 2B, does n refer to individual amyloplasts spread over multiple cells in one plant?
c. Figure 5B,C biological replicates also not clear
3. Statistics are not given for quantitative comparisons in Figure 2B,C and Figure 5B.
4. The authors make comparative statements about chloroplast and amyloplast division, but have not shown results regarding chloroplasts. Although these phenotypes are known in the literature, it would be good to explain those phenotypes more specifically when comparisons are made and perhaps also to compare at least some of them alongside the phenotypes of amyloplasts in the ovule integuments when direct comparisons are made. This way it is ensured that these differences are present under this particular experimental system. The authors also say 'we found no identical phenotypes between amyloplasts and chloroplasts in these mutants' (line 172, 173), but this statement is a bit vague because the WT phenotype between chloroplasts and amyloplasts is already different, so the comparison is not straightforward and should be elaborated.
5. Line 185 - data not shown for regular growth phenotypes - could this be added in the supplemental data? Same for amyloplast phenotypes.
6. Some statements that were a bit confusing:
a. Lines 257 - 260: 'The physiological significance of this stromule-localized FtsZ1 ring could not be directly measured, but the presence of stromules with a deep constriction (Supplemental Fig. S4C) and that of small plastids or amyloplasts (1-2 μm width) in mature integument cells (Supplemental Fig. S5) points towards successful stromule fission events.' Could the authors elaborate this statement? Were they not able to see stromule fission in live imaging? Could the small plastids also be underdeveloped plastids, rather than products of stromule fission?
b. Line 301: 'and explained why an FtsZ1 ring exists and functions preferentially at the stromule neck' - how do the dynamics of FtsZ1 rings at stromules explain this exactly?
c. Line 305 - 307: 'Their behavior (Supplemental Movie S1 and Fig. 4) indicated that these stromules were a product of giant amyloplasts rather than that of separate plastids.' - what is meant by this exactly? Is it that the stromules were associated with the edges of the giant amyloplast?
d. Line 313-314: The authors say that the phenotype of giant amyloplast-stromule associations in minE is a consequence of the increased elongation and stability of stromules, but it is unclear to me how the authors concluded this order of events.

e. The authors make the statement that the amyloplast division mechanism depends on the size of the amyloplast, but I don't really understand what they mean by this, as the amyloplast size would necessarily be influenced by amyloplast division.

Minor points

1. Line 182 and onwards, the authors refer to 'stroma-targeted fluorescent proteins' but it would be clearer if they elaborated here and in the figure legends that it was YFP, GFP and CFP with a transit peptide, and they should mention which transit peptide this is.
2. The authors sometimes mention 'plastids or amyloplasts' - it is not clear what is meant by this distinction. Clearer to just say one or the other?
3. Figure 2 - what is the difference between the blue and pink arrows?
4. Minor language corrections:

a. Line 71 - 'evolutional' should be 'evolutionary'
b. Line 136 - accumulate
c. Line 167, 168: change to: 'arc5, which has a mutation in the gene encoding for a dynamin related protein and has division arrested chloroplasts,'
d. Line 175: 'This thing' could be changed to 'this observation'

Response to Reviewers

Response to Reviewer #1

We are grateful to the helpful comments by Reviewer #1. Following these suggestions, we have revised the manuscript contents.

1. In our previous manuscript, we inferred that the rare occurrence of small plastids or amyloplasts in mature integument cells (Fig. S5 in the former version) may reflect stromule fission events by FtsZ ring. However, during the revision process, we noticed that the ftsZ triple mutant could also produce poorly developed plastids or amyloplasts in mature integument cells. In addition, the FtsZ ring formation in amyloplasts mainly occurs in their early developmental stage, as described in the manuscript (Fig. 3), and daughter plastids derived from stromules are expected to grow and start starch accumulation subsequently. Therefore, we straightforwardly examined the distribution of FtsZ ring in Phase I amyloplasts of the WT plants. We observed over 200 differentiating amyloplasts, and found that approximately 18% of FtsZ rings were located at stromules in our experimental condition. Based on these data, we have revised the Text and Fig. 3 accordingly. In addition, Supplemental Fig. S8 has been made to explain the experiment.

2. According to the advice by Reviewer #1, we performed DNA staining experiments using fixed ovules of *minE*. Developing ovules (Phase II) were excised from stromal CFP-expressing *minE* plants, fixed with 4%(w/v) paraformaldehyde, and stained with SYBR Green. By fluorescence microscopy, we obtained new data, including the presence of nucleoids in small plastids, the absence of nucleoids in many *minE* amyloplasts, and the rare but real presence of nucleoids in stromules. These results were summarized in a new Supplemental figure (Fig. S9).

3. Data of Table 1 have been presented in a graph (Fig. 1L) in the revised manuscript.

4. We have newly made Supplemental Fig. S2 to show slight variations in the amyloplast morphology in the WT plants. For comparison, microscopic images of chloroplasts are also presented.

5. The description has been improved to an appropriate one.

6. As Reviewer #1 suggested, the description has been corrected in order to avoid confusions.

7. The description has been improved, as our previous description suffered from over speculation.

8. The spelling of TGD has been revised.

9. We have corrected the wording.

10. The term ‘FtsZ1-ring’ has been replaced with ‘FtsZ ring’ throughout the manuscript.

Other than points described above, we have replaced several figure panels with better ones to present data clearly.

--

Reviewer #2 (Comments for the Author):

We thank Reviewer #2 for many constructive comments and advices. According to these suggestions, we have improved the previous version of our manuscript.

Major points

1.
a. Following an advice of Reviewer #1, we have changed Table 1 to Fig. 1L. By this change, a half population of amyloplasts in arc5 relative to WT have been clearly presented.

b. We have newly added Fig. 3C. In the WT, the number of the FtsZ ring in stromule-forming amyloplasts was mostly one. By comparing Fig. 3C and Fig. 3D,E, the increased frequency of FtsZ ring per plastid in the minE mutant became clear.

2.
a. In many amyloplast counting experiments, we recorded only the ovule and cell numbers, but not for the analyzed plants. Therefore, we performed additional amyloplast counting experiments using Col, Ler, Ws, arc5-1, arc11-1, atminE1-1, and ftsZ plants. As a result, combining with previous data, a total of 100 integument cells from 3 plants were investigated. Seeds of arc6-2 didn’t germinate in the lab. Since arc6-2 shares the identical nucleotide substitutions with arc6-1 and arc6-3 (Vitha et al., 2003), we examined arc6-1, instead of arc6-2, in the current revision. Data from 100 integument cells of arc6-1 have been incorporated into Fig. 1L.

b. Because we incorrectly described the term ‘plastid’ as ‘amyloplast’ in the previous manuscript, this point has been revised. ‘n’ refers to plastids. For both Fig. 2B and 2C, we performed additional experiments, and data from over 3 independent plants have been reorganized.

c. For Fig. 5B and 5C, we also conducted additional observations. As a result, data from a total of 3 independent plants have been obtained and reorganized.

3.
Statistical analyses have been performed on Fig. 2B and 2C (data are shown in Figs. S1 and S4) and Fig. 5B. Figs. 1L and 5C have been also assessed.

4.
According to helpful suggestions by Reviewer #2, we have newly made Supplemental Fig. S2. A variation of the plastid plan area (i.e., the size of amyloplasts (plastids) in the WT plants is presented in Fig. S1. In addition, we have improved descriptions in the previous descriptions (L172, 173) as suggested.

5.
Please understand that our data on the growth of crossed mutant plants (minD, minE, arc5 and arc6) were fragmentary, and during the revision process, seeds of some mutant lines in the lab did not germinate. To solve this situation, we would like to show that the ftsZ mutant exhibits relatively healthy growth and reproduction (Supplemental Fig. S10). For the amyloplast phenotype, we would like to show that Ws x Col (F1) and Ler x Col (F1) exhibited Col-like amyloplast phenotypes (Fig. 1M–O), in addition to the terminal phenotype of the mutant amyloplasts (Fig. S3).

6.
a. In the previous manuscript, we inferred that the rare occurrence of small plastids or amyloplasts in mature integument cells (Fig. S5) may reflect stromule fission events by FtsZ ring. However, during the revision process, we observed that the ftsZ triple mutant could also

produce poorly developed plastids or amyloplasts in mature integument cells. In addition, the FtsZ ring formation in amyloplasts mainly occurs in their early developmental stage (Fig. 3), and stromule-derived daughter plastids in the younger cell stages would be expected to grow and accumulate starch grains subsequently. Therefore, we straightforwardly examined the FtsZ ring distribution in Phase I amyloplasts of the WT plants. We investigated over 200 differentiating amyloplasts, and estimated that approximately 18% of FtsZ rings were located at stromules in our current experimental condition. In the research of plastid division, it is known that the final plastid fission stage is hard to follow in living cells, and this was true of the current microscopy of FtsZ1-GFP in stromules. Based on these data, we have revised the Text and Fig. 3 accordingly. Supplemental Fig. S8 has been newly made to explain the experiment.

b. The description has been improved.

c. The description has been improved.

d. At the initial stage (Phase I) of amyloplast development in minE, the cell size is relatively small and the stromule proliferation is also limited. At Phase II, the stromule development becomes remarkable in accordance with the cell expansion (Fig. 2). This, as well as the second-level observation of stromules (Fig. 4 and Supplemental Movies) supported the current notion.

e. The current notion of plastid size-dependent amyloplast division is related to our previous study on chloroplast MinD and MinE (Fujiwara et al., 2008, 2009). We previously hypothesized that as the plastids become larger, more constriction proteins would become necessary to enclose the organellar circumference. In the chloroplast division-site mutants, such as minD, smaller chloroplasts showed a tendency of division, whereas larger chloroplasts could not multiply even if FtsZ ring was initially made. In our current study, because the size of amyloplasts is small at Phase I, but it dramatically increases later. Therefore, it was considered that the growth of amyloplasts is an important factor that determines the process or fate of amyloplast division in minE.

Minor points

1. In this paper, we employed YFP, GFP and CFP as reporter proteins, which were fused with an N-terminal transit peptide from RbcS3A or FtsZ1. We also employed the full-length FtsZ1-GFP. To avoid possible confusions of readers by the use of various sequences, we would like to ask Reviewer's understanding for the simple designations of reporter fusions in this study.

2. In integument cells of *minE*, starch-containing amyloplasts and starchless plastids could co-exist. For this reason, we would like to ask Reviewer's permission to use this term case-by-case.

3. The arrowheads in Fig. 2 have been unified in cyan.

4. We thank all of these corrections. The manuscript has been revised.

Other than points described above, we have replaced several figure panels with better ones to present data clearly.

13-Mar-2024

Dr. Makoto T Fujiwara
Jochi Daigaku
Department of Materials and Life Sciences
Tokyo 102-8554
Japan

RE: Principles of amyloplast replication in the ovule integuments of Arabidopsis thaliana

Dear Dr. Fujiwara:

We have received reviews of your manuscript, which you may view at the end of this letter. On the basis of the advice received and my own evaluation, I believe that, pending the revision requests by the second reviewer, this article may ultimately be acceptable for publication in Plant Physiology.

When you are ready to submit your revision, click the below link and find the red arrow under your Author Tasks heading for this revision submission.

If you want to visit this version of the manuscript, you may click the link below and then click on the folder that reads "Post Decision Manuscripts".

Link Not Available

When uploading the revised version of this article, please be sure to include as complete an answer as possible to the referees' criticisms during the first round of reviews in the "response to reviewers" section.

Plant Physiology considers a revision for up to 60 days after a manuscript has been provisionally accepted; limited extension of this timeframe is at the discretion of the Monitoring Editor. A reminder notice will be sent to you 2 weeks before the deadline, at which time you may choose to contact the editorial office to request an extension; if so, please provide a justification for your request. After the expiration of this deadline, the article would need to be submitted as a resubmission and would be handled as such.

Thank you very much for giving us an opportunity to review this work. I look forward to receiving the next version.

Sincerely,

Alistair J. McCormick
Monitoring Editor, Plant Physiology

Want to add this revision deadline to your calendar? Click below!

-- Reviewer comments:
Reviewer #1 (Comments for the Author):

The authors have addressed almost all of the concerns I had regarding the previous MS. Although they have not shown the presence or absence of nucleoids in the wild-type small plastid that is generated by fission of stromules (this has been done for the minE mutant in the revised MS), it is probably technically demanding and can be addressed in the future. So, I believe the revised manuscript is publishable.

Reviewer #2 (Comments for the Author):

I thank the authors for the edits and additional data in the revision, and for addressing my comments. The manuscript is improved, but there are still points that need to be addressed properly before publication, mainly on clarification of the biological replicates and corresponding statistics.

1. The authors have added statistical analyses where they were missing previously, and have replotted and reanalysed some of their data. They now mention that their data comes from three independent plants, but in all of their analyses it is still not clear how this was taken into account in the statistical analysis.

For instance for Fig. 1L, did the authors pool all the cells from 3 independent plants to a total number of 100 and then do statistics as if there were 100 individual measurements? This would give an inaccurate evaluation of the statistical significance, because you actually have 3 biological replicates, not 100. This is probably why the p-value is so low. The cells that come from the same plant would be paired data, and for the statistics they cannot all be treated independently in a total pool of 100 independent data points, as this would be pseudoreplication. The analysis needs to be redone taking into account only 3 biological replicates, not 100. A nested model could be used for example. This is also the case for other figures - figure 2B, 2C and corresponding supplemental figures, and figure 5B, 5C. In addition to correcting the statistical analysis, it would also be helpful if the plotting differentiated the data from the 3 biological replicates (e.g. by using different colors).

I highlight some additional specifics related to these issues:

a. Fig 1: 'For each sample, 100 cells from several independent plants were observed' - The authors should also indicate in the legend that this data comes from 3 plants, not just 'several'.
b. In Figure S4, Phase III MinD only has 1 replicate? Doesn't seem statistically sound.
c. In Fig. 5B, do the dots represent individual plastids? The number does not seem equivalent to the number of plastids mentioned, it is not clear what the dots indicate.
d. L264 - 266: The authors make a statement about Figure S8 that over 200 differentiating amyloplasts were observed and that over 15% of FtsZ rings were in stromules, but do not show quantitative analyses.

2. A few additional points:

a. The extra data on nucleoids is interesting, but it would be helpful if the authors also included a WT for comparison, not just the minE mutant.
b. The authors explained in their response that they used both the terms 'amyloplast' and 'plastid' throughout the MS to distinguish between starch-filled and starchless plastids in the ovule integuments, but it would be helpful if they defined this more specifically at the start.
c. The authors mentioned that they wanted to keep the descriptions of the reporter lines simple in the text, but it would still be informative if they included the details about the transit peptides in the materials and methods.

Response to Reviewer #2

We thank Reviewer #2 for the helpful comments on the previous version of our manuscript. We have carefully checked the manuscript again and made further changes to improve the content.

1. According to the advice on statistical analyses provided by Reviewer #2, we conducted nested analyses using GraphPad Prism software. Figs. 1L, 5B, 5C, S1, and S4 in the previous version of the MS have been corrected, and two new figures (Figs. S1 and S12) have been added. Please understand that in the field of chloroplast research, the intracellular number of chloroplasts is generally reported on a 'cell number' basis (e.g., Pyke and Leech (1994) Plant Physiol. 104: 201-207; Marrison et al. (1999) Plant J. 18: 651-662; Okazaki et al. (2009) Plant Cell 21: 1769-1780). To our knowledge, high-resolution statistical analysis of individual Arabidopsis mutant plants has not been reported previously. Therefore, we would like to keep Figs 2B and 2C, which contain information extracted from Figs. S2 and S5, respectively, in their current forms.

a. We have corrected the Fig. 1 legend.

b. In this panel, we intended that only 1 of 100 amyloplasts (from three minD plants) had stromules. Two plants showed no stromules among the analyzed amyloplasts. This was taken into consideration in the nested one-way ANOVA in Fig. S5.

c. 'n = 50 plastids' has been described in the Fig. 5B legend.

d. The real number of FtsZ rings has been described in the text.

2.
a. We performed DNA staining of a WT plant (segregated from *minE* x TP-CFP cross). The data have been added to Fig. S10, and the text has been amended in the revised MS.

b. We have noted the use of the terms 'amyloplast' and 'plastid' in the text. We apologize if our explanations were insufficient in our previous response to this query. In the revised manuscript, we would like to use the term 'amyloplast' to refer to starch-containing plastids and 'plastid' as a general term for plastids, starchless plastids, and other not strictly defined plastids.

c. We have added information on the transit peptides in transgenic lines to the Materials and Methods section.

Other than the above, we have done our upmost to improve the previous version of the MS (e.g., the scale bar of Fig. S10 was corrected to 5 μm). In addition, the current MS has been proofread by a professional English edited service prior to submission. Please see the link to the editing certificate below.
https://www.bioedit.com/digital-certificate/?jobId=5D44633A-6239-4AD8-AC8E-C801C00C45D5&secret=abc

23-Apr-2024

Dr. Makoto T Fujiwara
Jochi Daigaku
Department of Materials and Life Sciences
Tokyo 102-8554
Japan

RE: Principles of amyloplast replication in the ovule integuments of Arabidopsis thaliana

Dear Dr. Makoto Fujiwara:

We are pleased to accept your manuscript for publication in the Plant Physiology. This acceptance is contingent on revision based on the comments of the reviewer below.

Please highlight all changes and include a detailed annotation to changes to the text, with line numbers, and noting your responses to the comments.

To submit your revised manuscript, click:

Link Not Available

If you cannot return the revised manuscript within 8 weeks of receipt, please let us know. Otherwise, we will assume that you have elected not to revise the manuscript and are withdrawing it.

Thank you for allowing us to review your work. We look forward to hearing from you soon.

Sincerely,

Alistair J. McCormick
Monitoring Editor, Plant Physiology

-- Reviewer comments:
Reviewer #2 (Comments for the Author):

I thank the authors for addressing the comments on the statistics and replicates, the supplemental figures with the individual replicates make the data much clearer. I just had a few points that would benefit from clearing up before final publication:

1. The authors need to better explain how they quantified the stromule length and frequency (Figure 2C and 5B, and supplemental figures), and what data are exactly shown in the figures. I am confused by the different labels in the figures and legends.

The legend says 'Mean length of stromules (n= 50 plastids for Phase II; indicated numbers (%) of plastids for Phase III) and their frequency (%) (n=150 plastids for Phase II and 100 plastids for Phase III)'. I am confused whether the frequency data and the stromule length data come from the same dataset. How was the stromule frequency determined? Are the numbers for Phase III referring both to absolute number of plastids and percentage of plastids with stromules because the total is 100? But how does this work for the Phase II data, if n=50 for stromule length but n=150 for frequency determination? Are the numbers for Phase II and Phase III showing different things? The numbers under Phase III seem to indicate the absolute number of plastids, according to figure S5 and S12. Is there only stromule frequency number for Phase II then? This is very confusingly represented if the numbers are referring to different things. I am confused what n=150 and n=100 are referring to, and what the stromule frequency is actually referring to. Is there information missing in these figures?

2. Figure 5
a. Plastid numbers for 5b ftsz and S12B ftsz don't seem to match up? Also in S12B, ftsz only has 2 replicates, is individual 3 misprinted?
b. In Figure 5C, authors mention that there are 4 individual plants used for minE proplastids, but in the accompanying figure S12C only three plants are mentioned - why is there this discrepancy?

3. The authors said in their response to my comment for the one MinD replicate in Figure S5: 'In this panel, we intended that only 1 of 100 amyloplasts (from three minD plants) had stromules. Two plants showed no stromules among the analyzed amyloplasts. This was taken into consideration in the nested one way ANOVA in Fig. S5.' It would be helpful if the authors made

this point clearer in the text/legend, see also comment 1 above regarding clarification of the data structure.

Response to Reviewer #2

We appreciate the comments of Reviewer #2 on the previous version of our manuscript. We understood that further improvements are necessary before the final form.

1. Fig. 2C, Fig. 5B, and Fig. S5, legends:
For measuring the stromule length, we used the dataset (fluorescence microscopy images) of stromule frequency analysis. However, not all the plastid images were appropriate for the measurement, because many stromules exhibited three dimensional growth in cells and those stromules were out-of-focus in the images. For this reason, we used up to 50 selected images of stromule-extending plastids for each analysis.

We have stated the definition of ‘stromule frequency’ and ‘stromule length’ in MATERIALS AND METHODS. In addition, we have improved legends of Fig. 2C and Fig. 5B to avoid confusions as Reviewer #2 suggested.

Lines 614, 669, and 678, and others:
Inconsistent labels or descriptions in figures and legends have been corrected.

2.
a. Fig. 5B and Fig. S12B, ftsZ data:
We apologize for the error of Fig. 5B, as 13 data points were found outside the axis limits or missing in the graph. ftsZ data in Fig. S12B is really from 3 plants (the disappearance of stromules could take place at late Phase III of ftsZ), but several data points were missing for ‘individual plant 2’. The graphs of Fig. 5B and Fig. S12B have been replaced with to correct ones. The layout of Fig. 5 has been also altered.

b. To start a nested one-way ANOVA using the software, we set three subcolumns for the number of analyzed plants. This initial setting was needed for multiple comparison tests.

3. Fig. S5.
We have added an explanation on the *minD* data to Fig. S5 legends. Also, we have defined the ‘stromule frequency’ and ‘stromule length’ in MATERIALS AND METHODS, as described above.

01-May-2024
Dr. Makoto T Fujiwara
Jochi Daigaku
Department of Materials and Life Sciences
Tokyo 102-8554
Japan

MSID: PP2023-RA-01597R3
MS TITLE: Principles of amyloplast replication in the ovule integuments of Arabidopsis thaliana

Dear Dr. Makoto Fujiwara:

I am pleased to inform you that your revised manuscript "Principles of amyloplast replication in the ovule integuments of Arabidopsis thaliana" has been accepted for publication in Plant Physiology. Your manuscript will be checked for consistency with journal formatting and image standards by one of our Science Editors, who will contact you shortly. They may have a number of queries and suggestions for you. Please keep in mind that they work to increase the online visibility of your article and its attraction for readers from the widest of backgrounds. You will have the opportunity to review any revisions, and I hope you will work constructively with them to get the most out of your research. You will also have the opportunity to review all changes when you receive the author proofs for your article.

Once validated for publication, your article will be sent to a Science Editor for a final review of your manuscript and source files. After your final files are uploaded, your article will appear in the next available issue and the online Preview version of your article will be posted after your signed Copyright and Checklist forms and final source files are received.

Please be sure to include the following in your manuscript file:
Harvard style citations
List of all author contributions and funding information at the beginning of manuscript after the titles and author list.
List all supplemental materials associated with the article, with titles, at the end of the ms, before the references and acknowledgments section.
Complete Figure Legends for all figures appearing in the article listed before the Literature Cited.

Thank you and congratulations on your Plant Physiology paper!

Sincerely,
Alistair J. McCormick
Monitoring Editor, Plant Physiology

=====
FIRST AUTHOR PROFILES
=====

We recognize the hard work that goes into being First Author of a Plant Physiology paper by publishing a "First Author Feature" for each First Author alongside your paper.

If the first author(s) would like to submit information for a feature on Facebook and Twitter, fill out the form by clicking on the link below:

Plant Physiology First Author Feature Form: https://forms.office.com/r/ZBCGZfhm8F
If you cannot access the form through the link above, please contact Rachel at rbelsky@aspb.org.

To upload an image of yourself to accompany your profile, please put your photo in the correct journal folder at the link below. You must label your image: Author First Name Last Name Article Title.

First Author Profile Image Dropbox: https://www.dropbox.com/scl/fo/xz029p7qjr2jml2rp2kmq/h?dl=0&rlkey=6fkxcaqmxx35ap152b2osy1fz

=====

IMPORTANT REMINDER: PEER REVIEW REPORTS
=====

If you opted to publish a peer review report along with your article during the original submission process, it will be prepared by the editorial staff and publicly posted with your manuscript, inside the zip file that contains any other supplemental material. As a reminder, the peer review report is a public record of all comments from editors and reviewers, as well as your prior responses, as you received them in the decision letters for each draft of your manuscript. If you agreed to publish this report and have changed your mind, or are not sure if you selected this option, please contact the editorial office as soon as possible before signing the license agreement from our publisher.

-- Reviewer comments:
